# Endoplasmic reticulum contact sites regulate the dynamics of membraneless organelles

**DOI:** 10.1126/science.aay7108

**Published:** 2020-01-31

**Authors:** Jason E. Lee, Peter I. Cathey, Haoxi Wu, Roy Parker, Gia K. Voeltz

**Affiliations:** 1 Department of Molecular, Cellular, and Developmental Biology, University of Colorado, Boulder, CO, USA.; 2 Howard Hughes Medical Institute, University of Colorado, Boulder, CO, USA.; 3 Department of Biochemistry, University of Colorado, Boulder, CO, USA.

## Abstract

**INTRODUCTION:**

The cytoplasm contains an unconventional class of organelles that concentrate specific factors and resources without a limiting membrane. These membraneless organelles include ribonucleoprotein (RNP) granules such as processing bodies (P-bodies, or PBs) and stress granules. PBs and stress granules are composed of nontranslating messenger RNAs (mRNAs) and associated proteins and are thought to provide discrete biochemical environments for regulating the translation and/or degradation of mRNA. In contrast to membrane-bound organelles, very little is known about what extrinsic and intrinsic factors regulate the fusion and fission of membrane-less organelles. Recently, an unexpected role for the endoplasmic reticulum (ER) has been observed in regulating the biogenesis of other membrane-bound organelles at contact sites where the two organelles are tethered and closely apposed. ER contact sites can allow the direct exchange of macromolecules and serve as a platform for the recruitment of machineries that regulate organelle biogenesis, division, and trafficking. Here, we found that ER contact sites can also regulate the biogenesis and fission of two types of membraneless organelles, PBs and stress granules.

**RATIONALE:**

To determine the extent to which PBs, a conserved cytoplasmic membraneless organelle, are tethered to the ER in animal cells, we used live-cell fluorescence microscopy to simultaneously track the spatiotemporal dynamics of the ER and PBs. To overcome the diffraction limits associated with light microscopy, we designed a reversible ER-PB contact assay using probes attached to the ER and PBs that emit a high-intensity fluorescence signal when the probes are close enough to dimerize. Because ER morphology and RNP granule biogenesis are tightly linked to mRNA translation, we systematically evaluated the relationships between ER morphology, RNP granule biogenesis, and mRNA translation by assessing endogenous PB numbers in response to altering ER shape and translational capacity and to the induction of cytosolic and ER stress. Because PBs and stress granules are dynamic organelles that undergo fission and fusion reactions akin to membrane-bound organelles, we used live-cell fluorescence microscopy to score the spatiotemporal relationship between the position of RNP granule division and contact sites with ER tubules.

**RESULTS:**

Using multiple measures, we found that a population of PBs were tethered to the ER in human cells. ER shape exerted profound effects on PB numbers and PB-ER contact. Conditions that promoted expansion of peripheral ER tubules and a reduction in peripheral ER cisternae increased PB numbers and ER-PB contact. Conversely, conditions that promoted an expansion of ER cisternae dramatically decreased PB numbers. The effect of ER shape on PB abundance was likely a reflection of the relative translational capacity of the ER domains. Owing to differences in ribosome density, smooth ER tubules are presumed to have a lower translational capacity than rough ER cisternae. Conditions that locally enhanced the translational capacity of the ER by increasing ER cisternae, such as ER stress, also reduced the number of PBs. Conversely, conditions that globally inhibited mRNA translation (NaAsO_2_ and puromycin) suppressed the effects of ER shape on PB abundance. Thus, ER contact sites affected the proliferation of PBs under basal and translationally repressed conditions. Furthermore, ER contact sites also affected the mysterious PB fission process. Live-cell imaging revealed that dynamic ER tubules define the position where PB and stress granule division occurs. These data mirror the spatiotemporal role of ER tubule contact domains that drive the constriction and division of membrane-bound organelles like endosomes and mitochondria.

**CONCLUSION:**

Here, we found that the ER contains contact site domains that are capable of tethering both membraneless and membrane-bound organelles. ER structure and translational capacity has effects on PB biogenesis. Furthermore, the fission of cytoplasmic RNP granules appears to represent an active process that can be driven by ER contact sites, analogous to the division of membrane-bound organelles.

**Endoplasmic reticulum tubules are a component of the ribonucleoprotein granule fission machinery.:**

Membraneless RNP granules undergo fission and fusion similar to membrane-bound organelles. A cartoon (top) and the corresponding live-cell fluorescent images (bottom; at 0−, 5−, and 10-s time points, from left to right) of a PB (green) undergoing division at a position where an ER tubule is crossing (red).

Tethered interactions between the endoplasmic reticulum (ER) and other membrane-bound organelles allow for efficient transfer of ions and/or macromolecules and provide a platform for organelle fission. Here, we describe an unconventional interface between membraneless ribonucleoprotein granules, such as processing bodies (P-bodies, or PBs) and stress granules, and the ER membrane. We found that PBs are tethered at molecular distances to the ER in human cells in a tunable fashion. ER-PB contact and PB biogenesis were modulated by altering PB composition, ER shape, or ER translational capacity. Furthermore, ER contact sites defined the position where PB and stress granule fission occurs. We thus suggest that the ER plays a fundamental role in regulating the assembly and disassembly of membraneless organelles.

## Graphical Abstract

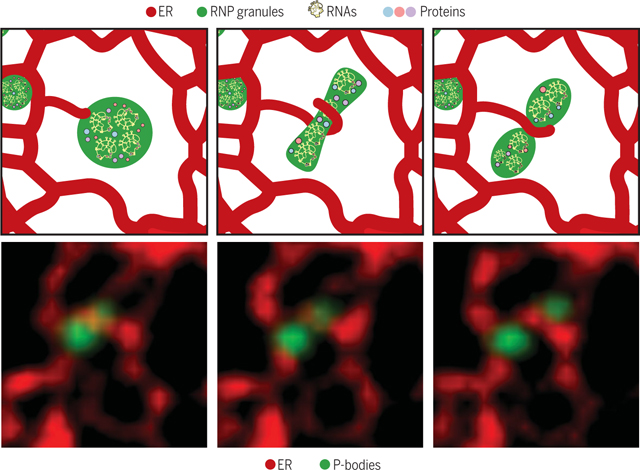

The endoplasmic reticulum (ER) forms membrane contact sites (MCSs) with other membrane-bound organelles to control their composition and distribution throughout the cell (1, 2). MCSs provide an alternative to vesicle-based interorganelle exchange by forming a conduit between two organelles that allows for rapid exchange of resources like lipids and calcium (1–7). ER-organelle MCSs also provide a platform for the recruitment of machineries that regulate bidirectional organelle trafficking on microtubules and help to define the position of organelle division (8–14). In addition to membrane-bound organelles, the cytoplasm is further compartmentalized through condensation of cytosolic macromolecules into a variety of membraneless organelles that differ in function, composition, and number (15, 16). Because the biogenesis and maintenance of membrane-bound organelles are controlled by ER MCSs, we speculated that the dynamics of membraneless organelles might also be regulated by an additional class of ER contact sites.

Ribonucleoprotein (RNP) granules, such as processing bodies (P-bodies, or PBs) and stress granules, are membraneless organelles with distinct structures that are conserved from yeast to animal cells (15, 16). PBs and stress granules are formed from translationally inactive pools of messenger RNAs (mRNAs) and associated proteins and exclude the translation machinery (17). PBs are constitutive structures that are enriched for specific and proteins involved in mRNA silencing, decapping, and decay (18–21). Conversely, stress granules are transient structures that form when translation is restricted (22). Stress granules are enriched with RNA binding proteins and some translation initiation factors and contain most mRNAs with a bias toward enriching for longer mRNAs (23, 24). Thus, PBs and stress granules appear to be long- and short-term holding sites for mRNAs poised to be released into the translation pathway depending on cytoplasmic cues like metabolism and translation capacity. Given the ER-localized translation of mRNA encoding proteins that enter the secretory pathway (25–27) and the stress-dependent release of ER-localized mRNA (28), there could also be RNP granules that store mRNAs for translation reinitiation at the ER membrane.

## PB dynamics are coupled to ER tubule dynamics in human cells

**Fig. 1.**
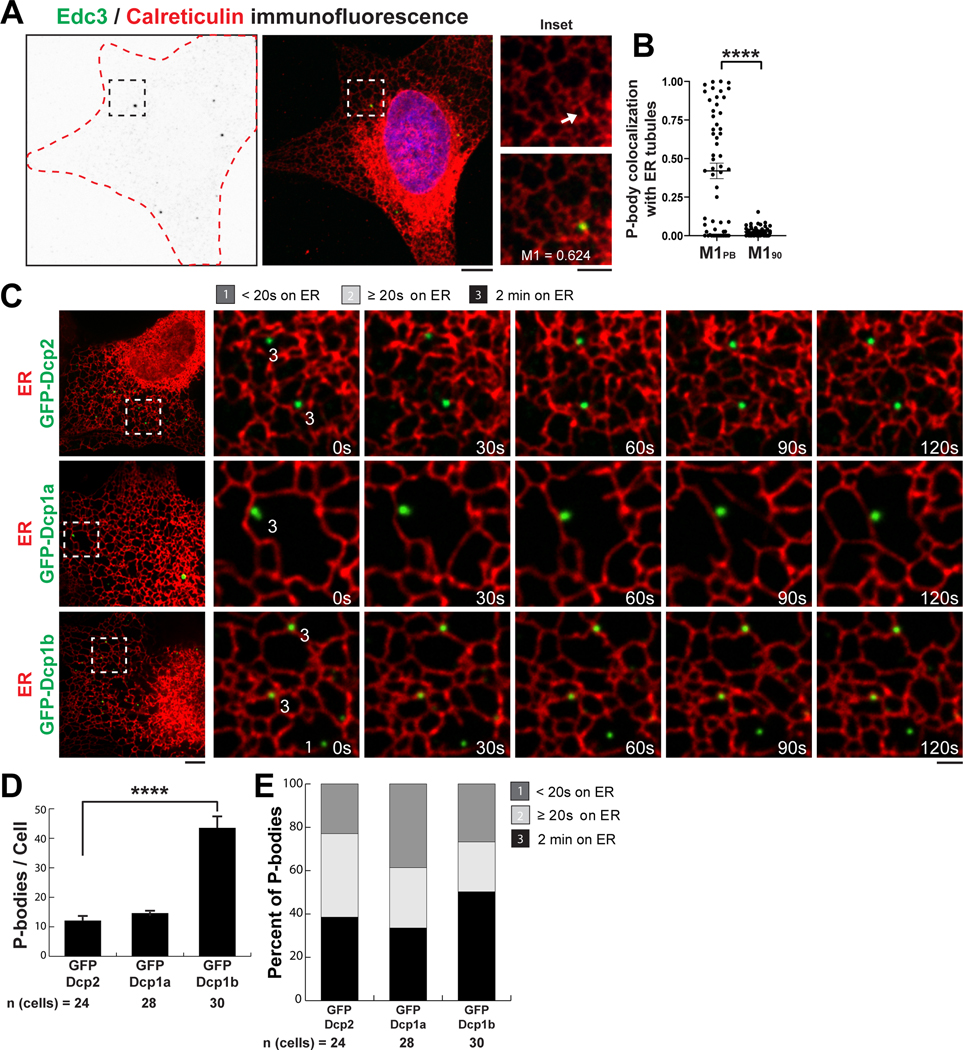
A subset of PBs colocalize and track with ER tubules in human cells. (**A**) Representative images of immunofluorescence (IF) studies performed in U-2 OS cells against Edc3 and calreticulin to label PBs (green) and ER (red), respectively. Edc3 IF gray-scale images were inverted to highlight Edc3 puncta (left), Edc3 IF in green merged with the nuclear stain Hoechst in blue (middle), and the middle panels merged with calreticulin IF in red (right). (**B**) The level of colocalization between endogenous PBs and ER tubules was determined by calculating the Mander’s coefficient of PBs within regions of interest (ROIs) with resolvable ER tubules (M1_PB_) and tested for significance using the Kolmogorov-Smirnov test by comparing M1_PB_ to the Mander’s coefficient after the ER ROI was rotated 90° (M1_90_). Fifty-six ROIs were analyzed out of 50 cells from three biological replicates. The error bar indicates SEM. (**C**) Representative merged images of the ER (mCh-KDEL) and PBs labeled with three factors of the decapping complex (GFP-Dcp2, GFP-Dcp1a, or GFP-Dcp1b). Insets show movement of the two organelles through space and time over a 2-min time-lapse movie with frames captured every 5 s (Movie 1). PBs labeled with a “1” tracked with ER tubules for less than four consecutive frames, whereas PBs labeled with a “3” tracked with ER tubules for the entire time-lapse movie. (**D** and **E**) Thirty cells were imaged for each condition from three biological replicates and quantified for the mean number of PBs per cell (D) and the degree of association between the ER and PBs (E). For (D), statistical significance was determined by one-way analysis of variance (ANOVA) with multiple comparisons, and error bars indicate SEM. For (A) and (C), scale bars are 5 and 2 μm in the full cell and inset images, respectively. ****P < 0.0001.

PBs are an ideal membraneless organelle to begin studies on the interface between the ER and membraneless organelles because PBs are constitutively present in all tested interphase cells and because PBs can grow, shrink, fuse, and undergo fission akin to membrane-bound organelles (17, 29). Two lines of evidence obtained in the budding yeast *Saccharomyces cerevisiae* suggest that PB biogenesis could be regulated by ER contact sites. First, immunogoldlabeled PBs have been observed adjacent to the ER in electron micrographs, and second, PB factors can sediment with the ER in sucrose gradients (19, 21). We thus set out to test the hypothesis that PBs are tethered to the tubular ER network in animal cells using immunofluorescence analysis. A human osteosarcoma (U-2 OS) cell line was fixed and immunolabeled with antibodies against a general luminal ER protein (calreticulin, red) and a PB component [enhancer of decapping (Edc3), green] (Fig. 1, A and B). The colocalization of PBs over the ER network was measured by Mander’s coefficient. A large subset of PBs colocalized with ER tubules. As a control, we rotated the ER image 90° and could then see that only a small percentage of PBs overlapped with the ER by chance (Fig. 1, A and B). Thus, a population of endogenous PBs may be bound to the ER network in animal cells.

We next used live-cell imaging to examine the extent to which PBs remained tethered to the ER over time, similar to experiments done to show that the ER is tethered to membrane-bound organelles (9). The tubular ER network is very dynamic. Thus, sustained contact between an individual PB and ER tubules over time suggests that the two organelles are tethered to each other. We collected 2-min movies of U-2 OS cells transiently transfected with fluorescent markers for the ER (mCh-KDEL) and three separate PB markers (GFP-Dcp2, GFP-Dcp1a, and GFP-Dcp1b; GFP, green fluorescent protein) (Fig. 1C, Movie 1, and fig. S1). PB contact with the ER was binned into three categories: The PB contacts the ER for (i) less than 20 s or not at all, (ii) at least 20 s but less than 2 min, and (iii) the entire 2-min movie.

Approximately one-third to one-half of exogenously tagged PBs stably associated with the ER throughout the 2-min movie depending on whether GFP-Dcp2 (39.1 ± 3.9%), GFP-Dcp1a (33.5 ± 5.0%), or GFP-Dcp1b (50.3 ± 4.7%) was expressed (Fig. 1E). By comparison, cells overexpressing GFP-Dcp1b contained fourfold more PBs (~40 PBs per cell, Fig. 1D), and this led to an increase in the percentage of PBs that were associated with the ER (80.4 ± 3.1%) (fig. S1, A to C). Thus, a substantial subset of PBs are tethered to the ER, and PB-ER contact is sensitive to PB composition and abundance.

## Nanoscale resolution of ER-PB contact using reversible dimerization-dependent fluorescent proteins in living cells

**Fig. 2.**
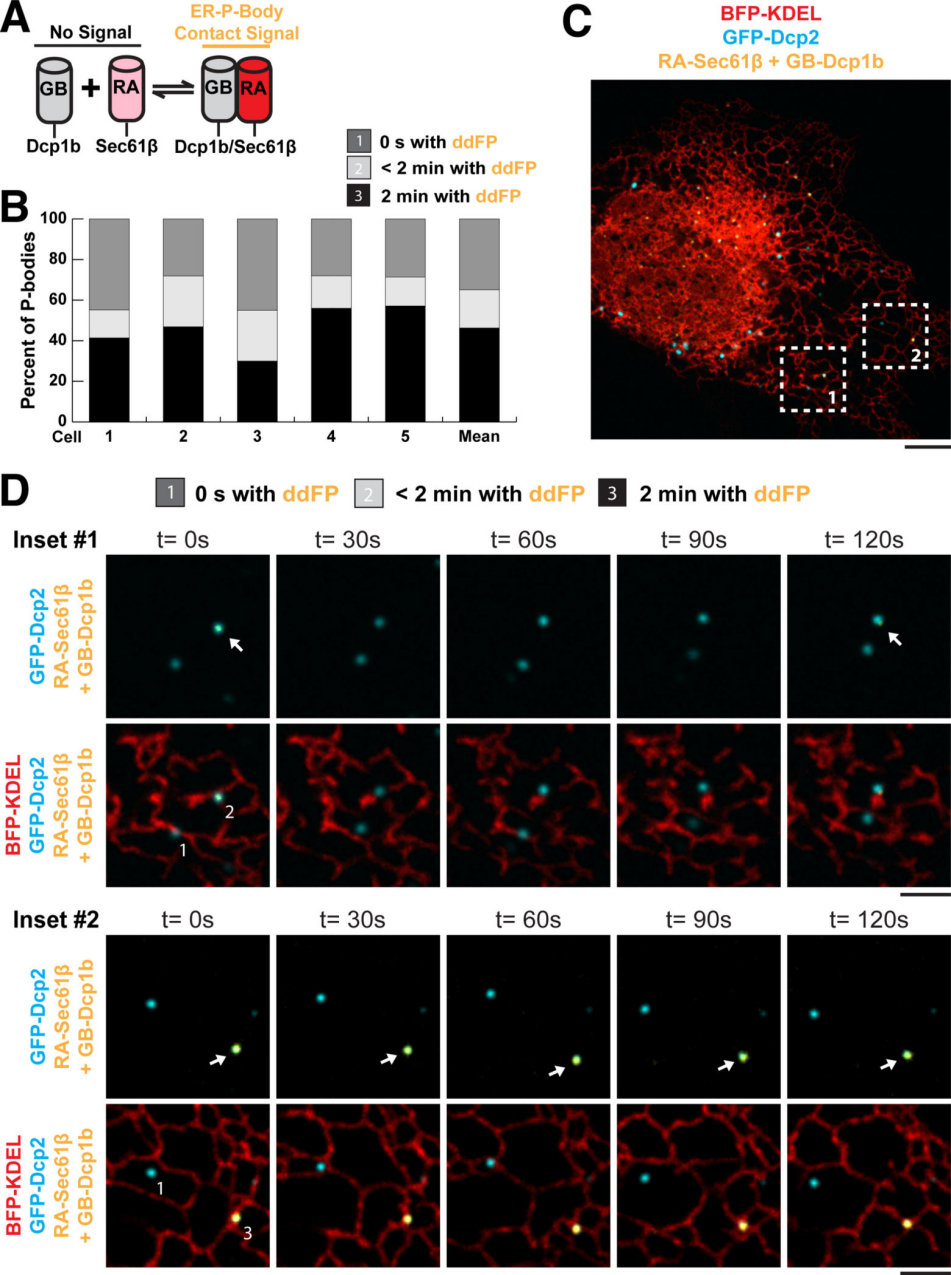
The ER forms transient and stable contact sites with PBs. (**A**) Cartoon of ddFP system used to assay ER (RA-Sec61β) and PB (GB-Dcp1b) contact. (**B**) Quantification of ER contact from five cells together with their mean values (*n* = 113 PBs). PBs were binned into three categories: (i) PBs without ddFP signal, (ii) PBs with ddFP signal for a fraction of the 2-min time-lapse movie, and (iii) PBs with ddFP signal for the entire 2-min time-lapse movie. (**C**) Representative merged image of Dcp2-marked PBs (cyan), KDEL-labeled ER (red), and ER contact (yellow). The numbered regions indicate the inset areas shown in (D). (**D**) Inset 1 (Movie 2) and inset 2 (Movie 3) show examples of each PB category. The arrows highlight time points with positive ddFP signal (yellow). Scale bars are 5 μm in (C) and 2 μm in (D).

The live-cell tracking of PBs with ER tubules over time strongly suggested that the two organelles are tethered. Because previous descriptions of RNP granules suggest that they are surrounded by a liquid phase (30, 31), we aimed to test whether ER tubules contact PBs at molecular distances that are reminiscent of typical MCSs (10 to 30 nm) (1, 2). Because 30 nm is below the resolution limit of our microscope, we used dimerization-dependent fluorescent protein (ddFP) domains (32) to resolve molecular contact between the ER and PBs in live cells (Fig. 2A). We fused the core (GB) domain to a PB marker (Dcp1b) and the red fluorescence–capable (RA) domain to an ER marker (Sec61β) such that red fluorescence will signal that the two organelles are close enough for the two tags to dimerize (Fig. 2A). The ddFP system is attractive for assessing contact sites in live cells because the interactions between ddFP domains are reversible (32). We captured 2-min movies of U-2 OS cells exogenously expressing PB (GFP-Dcp2) and ER (BFP-KDEL; BFP, blue fluorescent protein) markers together with the ddFP system and binned PBs into three categories: (i) PBs with no ddFP signal, (ii) PBs with ddFP signal for at least one frame of the 2-min movie, and (iii) PBs with ddFP signal for the entire 2-min movie (Fig. 2, C and D, and Movie 2 and 3).

Nearly half of the PBs maintained stable contact sites with the ER through the duration of the movie (46.3 ± 5.0%) (Fig. 2B). The frequency of stable PB-ER contact sites was similar to the qualitative tracking of two organelles in live-cell movies (Fig. 1E). The PBs in category (ii) also revealed that PBs can be recruited to and released from the ER (Fig. 2D, inset 1, and Movie 2).

## PB biogenesis is dependent on ER morphology and the translational capacity of the ER

PB and stress granules store translationally inactive mRNAs (15–17). The rough ER is bound by ribosomes and is a major site of translation, translocation, and protein folding in the cell (25–28). Electron microscopy and tomography have revealed that cisternal ER sheets have approximately fivefold higher ribosome density than ER tubules (33, 34). Conversely, ER tubules are functionally linked to phospholipid and sterol synthesis, calcium homeostasis, and the formation of ER-organelle MCSs (1, 2). Because rough cisternal ER is indicative of ER translational capacity, we tested whether altering peripheral ER shape would affect PB biogenesis.

**Fig. 3.**
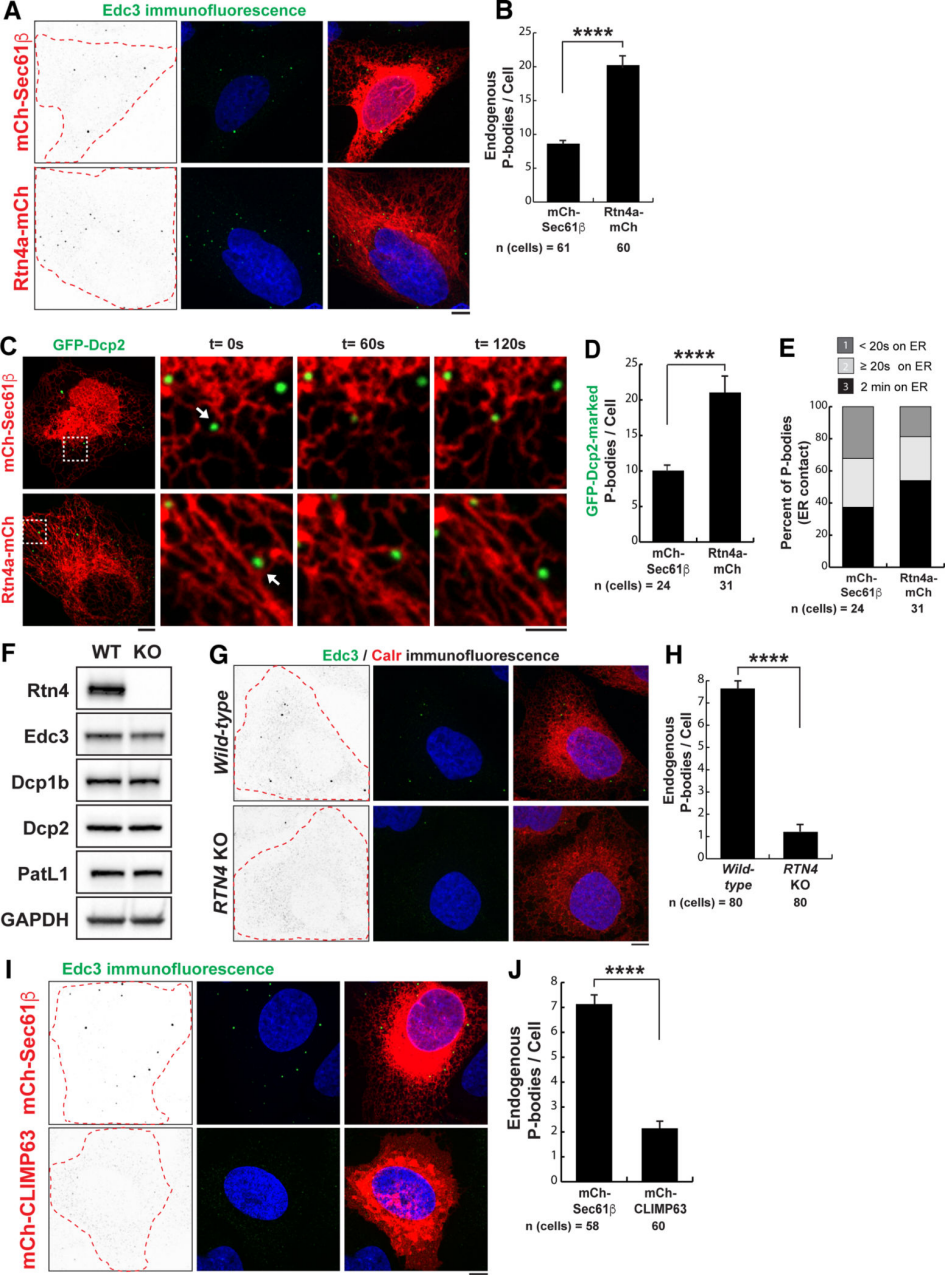
The relationship between ER shape and PB biogenesis. (**A**) Representative images of Edc3 IF studies performed in U-2 OS cells transiently transfected with mCh-Sec61β or Rtn4a-mCh. Edc3 IF gray-scale images were inverted to highlight Edc3 puncta (left), Edc3 IF in green merged with the nuclear stain Hoechst in blue (middle), and the middle panels merged with mCh-tagged ER markers in red (right). The dashed lines indicate the cellular boundary as estimated by the outer reaches of ER signal. (**B**) The mean quantified in U-2 OS cells exogenously expressing mCh-Sec61β or Rtn4a-mCh from three biological replicates. (**C**) Representative merged images of U-2 OS cells overexpressing either mCh-Sec61β or Rtn4a-mCh together with the PB marker GFP-Dcp2. Insets show movement of the two organelles through space and time over a 2-min time-lapse movie with frames captured every 5 s. PBs were binned as in Fig. 1C. The arrows show PBs that maintain contact with the ER for the entire movie. (**D** and **E**) Cells were imaged for each condition from three biological replicates and quantified for the mean number of PBs per cell (D) and the degree of association between the ER and PBs (E). (**F**) Immunoblot analyses of protein expression levels for Rtn4 and PB factors (Edc3, Dcp1b, Dcp2, and PatL1) in whole-cell lysates of wild-type and *RTN4* KO U-2 OS cells. Glyceraldehyde-3-phosphate dehydrogenase (GAPDH) was used as a loading control. (**G**) Representative images of IF studies performed in wild-type and *RTN4* KO U-2 OS cells against Edc3 and calreticulin (Calr) to label PBs (green) and ER (red), respectively. Edc3 IF gray-scale images were inverted to highlight Edc3 puncta (left), Edc3 IF in green merged with the nuclear stain Hoechst in blue (middle), and the middle panels merged with calreticulin IF in red (right). (**H**) The mean numbers of PBs were quantified in wild-type and *RTN4* KO U-2 OS cells from three biological replicates. (**I**) Representative images of Edc3 IF studies performed in U-2 OS cells transiently transfected with mCh-Sec61β or mCh-CLIMP63. Edc3 IF gray-scale images were inverted to highlight Edc3 puncta (left), Edc3 IF in green merged with the nuclear stain Hoechst in blue (middle), and the middle panels merged with mCh-tagged ER markers in red (right). (**J**) The mean numbers of endogenous PBs were quantified in U-2 OS cells exogenously expressing mCh-Sec61β or mCh-CLIMP63 from three biological replicates. In (B), (D), (H), and (J), statistical significance was determined by Student’s *t* test, and error bars indicate SEM; ****P < 0.0001. In (A), (C), (G), and (I), scale bars are 5 and 2 μm in full cell and inset images, respectively.

**Fig. 4.**
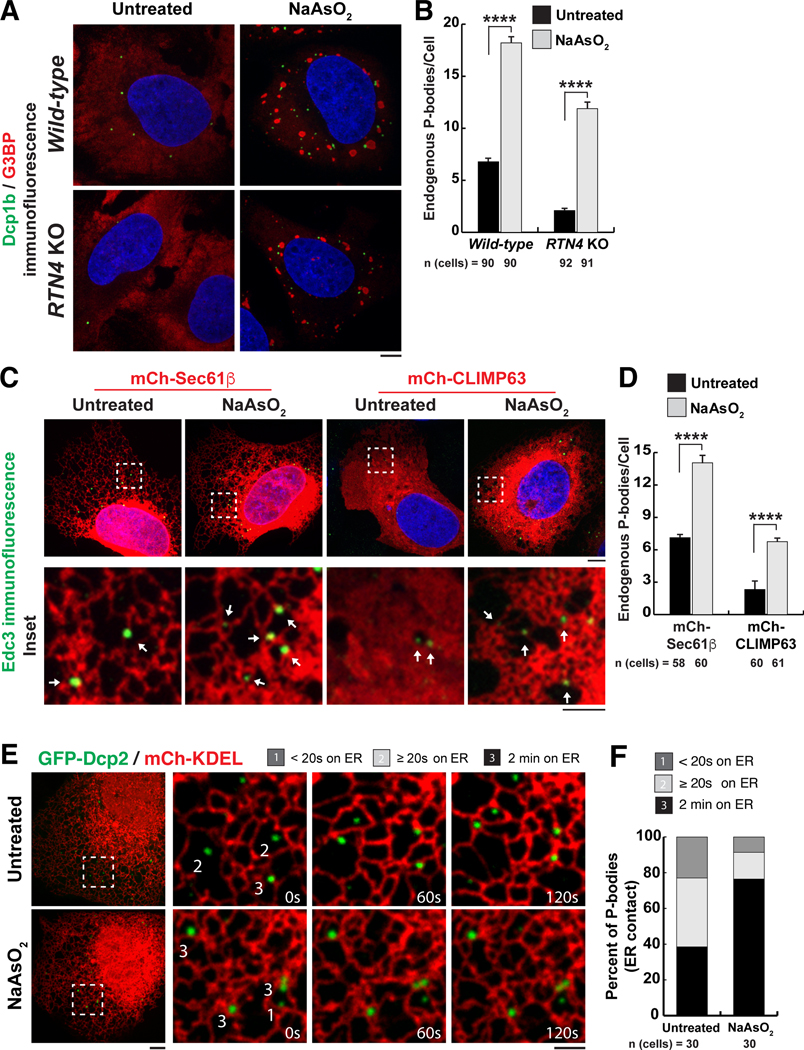
The relationship between PB biogenesis, ER shape, and oxidative stress–induced mRNA translation inhibition. (**A**) Representative images of Dcp1b (PB marker, green) and G3BP (stress granule marker, red) IF studies were performed in wild-type and *RTN4* KO U-2 OS cells treated with 0.5 mM NaAsO_2_ for 1 hour. Blue indicates the nucleus stained by Hoechst. (**B**) The mean numbers of endogenous PBs were quantified from three biological replicates. (**C**) Representative images of Edc3 IF studies performed in U-2 OS cells transiently transfected with mCh-Sec61β or mCh-CLIMP63 treated with 0.5 mM NaAsO_2_ for 1 hour. Arrows within insets highlight PBs that overlap with domains of high ER membrane curvature. (**D**) The mean numbers of endogenous PBs were quantified from three biological replicates in U-2 OS cells exogenously expressing mCh-Sec61β or mCh-CLIMP63 that were either untreated or treated with NaAsO_2_. (**E** and **F**) Representative merged images of U-2 OS cells exogenously expressing GFP-Dcp2 and mCh-KDEL to label PBs and the ER in live cells, respectively (E). Insets show movement of the two organelles through space and time over a 2-min time-lapse movie with frames captured every 5 s. PBs were binned as in Fig. 1C. Cells were imaged for each condition from three biological replicates and quantified for the degree of association between the ER and PBs (F). In (B) and (D), statistical significance was determined by one-way ANOVA with multiple comparisons, and error bars indicate SEM; ****P < 0.0001. In (A), (C), and (E), scale bars are 5 and 2 μm in full cell and inset images, respectively.

First, ER tubules were increased at the expense of cisternal ER by overexpressing the ER tubule–generating protein reticulon-4a (Rtn4a) (35), which led to a twofold increase in PBs compared with that in control cells expressing a general ER membrane marker, mCh-Sec61β (Fig. 3, A and B). Converting cisternal ER into tubules also led to an increase in ER-PB contact (Fig. 3, C to E). Next, we performed *RTN4* gene ablation by CRISPR-Cas9 in U-2 OS cells to shift the balance of cisternae and tubules toward more cisternal ER. *RTN4* knockout (KO) cells displayed significantly fewer PBs compared with wild-type U-2 OS cells, even though they expressed PB factors at similar levels to wild-type U-2 OS cells (Fig. 3, G and H). We confirmed that the *RTN4* KO phenotype was due to a loss in Rtn4a function because PB biogenesis could be rescued by exogenous expression of Rtn4a-mCh into *RTN4* KO cells (fig. S2). Finally, we depleted ER tubules by overexpressing the cisternal ER promoting protein CLIMP63 (36), which led to a significant decrease in PB numbers, reminiscent of *RTN4* KO (Figs. 3, I and J, and 4, C and D). Thus, ER structure affects PB biogenesis in a way that is likely reflective of the translational capacity of the ER.

## Inhibition of mRNA translation overrides the dependence of PB biogenesis on ER morphology

We next tested whether the dependence of PB biogenesis on ER shape is due to altering ER translational capacity. We restricted translation by inducing an oxidative stress response under cisternal ER-promoting conditions using sodium arsenite (NaAsO_2_), which induces a well-studied translation inhibition and RNP granule biogenesis response (17). We observed a significant increase in PB and stress granule abundance in wild-type cells after NaAsO_2_ treatment for 60 min (Fig. 4, A and B). PB abundance also increased significantly after NaAsO_2_ treatment under two cisternal ER-promoting conditions, *RTN4* depletion (Fig. 4, A and B) or mCh-CLIMP63 overexpression (Fig. 4, C and D). Furthermore, a closer inspection of PBs induced by NaAsO_2_ revealed a localization to ER domains of high membrane curvature, such as tubules, fenestrae, and edges of cisternae (Fig. 4C, insets). Thus, we measured the effect of NaAsO_2_ treatment on the percentage of PBs that are bound to ER tubules. ER-PB contact was tracked in live cells expressing GFP-Dcp2 and mCh-KDEL. Translation inhibition induced by NaAsO_2_ treatment doubled the percentage of PBs that were tightly associated with the ER in these cells compared with untreated cells (Fig. 4, E and F).

We aimed to complement NaAsO_2_-induced RNP granule studies with puromycin to directly target the translation machinery in wild-type and *RTN4* KO cells. Puromycin is an antibiotic that inhibits global translation by disrupting peptide transfer, leading to the release of ribosomes from mRNA. We used a short 15-min treatment and high 200 μM concentration of puromycin that can clear ribosomes off ER membranes (36). The short puromycin treatment also allowed us to minimize any downstream effects that accompany a global translation block.

We observed a significant increase in PB numbers in response to puromycin-induced release of ribosomes from mRNA and ER membranes in both wild-type and *RTN4* KO cells (fig. S3). As expected (22), puromycin did not induce stress granule formation. Thus, the relationship between PB biogenesis and ER shape is dependent on the translational capacity of the ER because inhibiting mRNA translation with NaAsO_2_ or puromycin could induce PB biogenesis even when cisternal ER is abundant.

## ER stress and the unfolded protein response induce PB disassembly

**Fig. 5.**
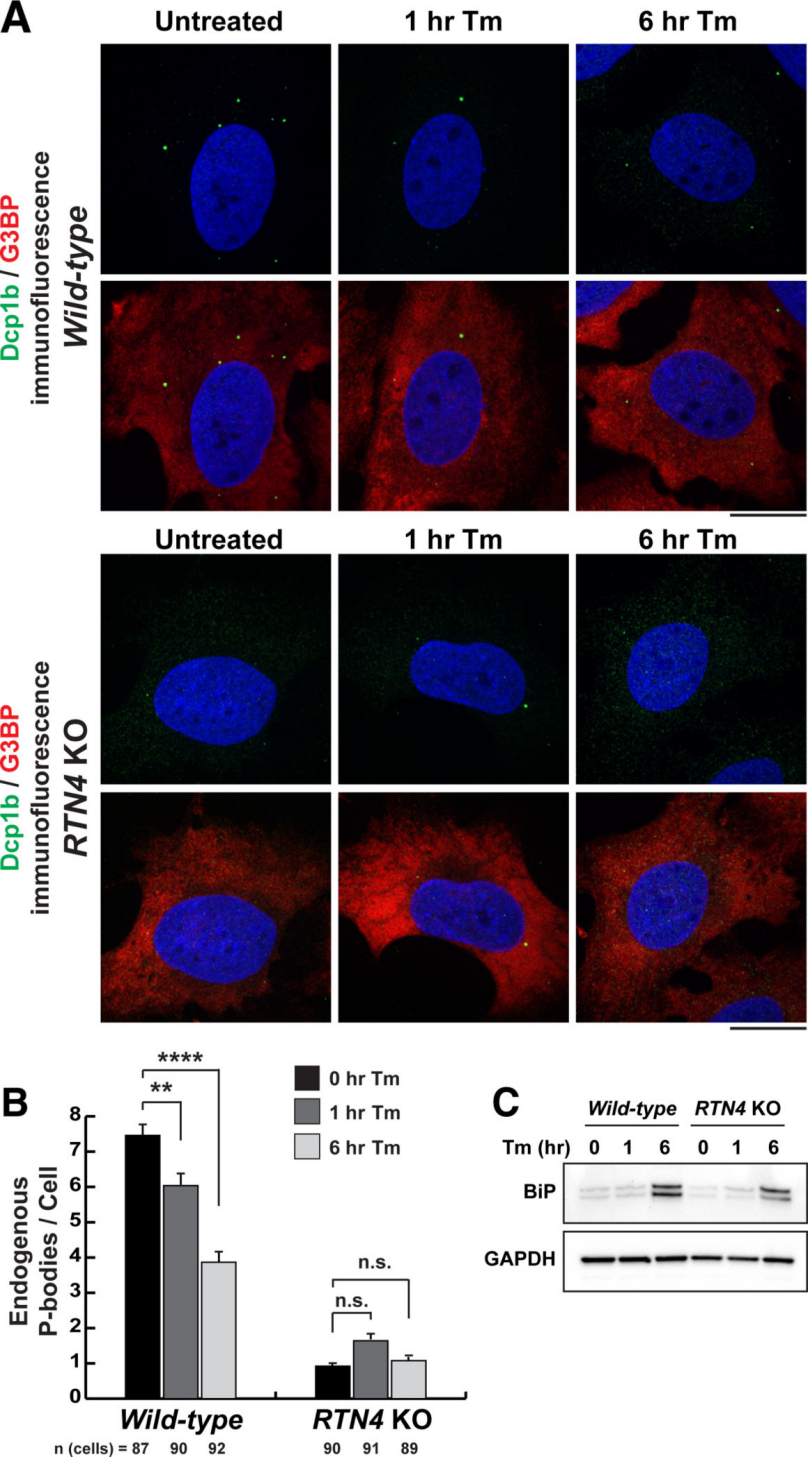
ER stress and the UPR triggers PB disassembly. (**A**)Representative images of Dcp1b (PB marker, green) and G3BP (stress granule marker, red) IF studies were performed in wild-type and *RTN4* KO U-2 OS cells that were either left untreated (0 hours) or treated with 1 μg/ml Tm for 1 and 6 hours. Scale bars are 5 μm. (**B**) The mean numbers of endogenous PBs were quantified from three biological replicates. Statistical significance was determined by one-way ANOVA with multiple comparisons, and error bars indicate SEM; n.s. is not significant, **P < 0.01, and ****P < 0.0001. (**C**) Immunoblot analyses of protein expression levels for the ER chaperone BiP were performed in whole-cell lysates of wild-type and *RTN4* KO U-2 OS cells treated with 1 μg/ml Tm to verify induction of the UPR. GAPDH was used as a loading control.

Cisternal ER and ER translational capacity will also increase when the unfolded protein response (UPR) is evoked during ER stress. The UPR can relieve the burden of misfolded protein accumulation by attenuating general translation and by selectively up-regulating and translating genes encoding for ER resident proteins such as chaperones (37). Thus, we further tested the relationship between ER shape, ER translational capacity, and PB biogenesis by inducing the UPR. Cells were treated with tunicamycin (Tm), which triggers the UPR by blocking N-linked glycosylation of nascent proteins in the ER lumen. Wild-type and *RTN4* KO cells were treated with Tm for 1 and 6 hours to resolve any differences between acute pre-UPR and UPR conditions (Fig. 5). We confirmed that the UPR was induced by 6-hour Tm treatment by detecting increased levels of the ER chaperone BiP and expansion of cisternal ER (Fig. 5C and fig. S4).

Induction of the UPR by Tm reduced PB numbers in wild-type cells and had no measurable effect on the already low PB numbers in *RTN4* KO cells (Fig. 5, A and B). Surprisingly, Tm-induced UPR did not trigger stress granule formation in either wild-type or *RTN4* KO cells (Fig. 5A). However, previous studies connecting ER stress to stress granules did so by using thapsigargin, which has effects that extend beyond ER stress owing to the release of calcium from the ER into the cytosol (38).

Taken together, our data show a consistent relationship whereby conditions that increase cisternal ER or ER translational capacity reduce PB numbers, and conversely, conditions that decrease cisternal ER or translational capacity increase PB numbers and increase ER-PB contact. Given that PBs store and degrade translationally inactive mRNAs, the ability of PBs to form and disassemble in response to changes in ER translational capacity opens up the possibility that ER-PB contact sites are conduits for mRNA exchange between the two organelles.

## ER tubules mark the sites of RNP granule fission

**Fig. 6.**
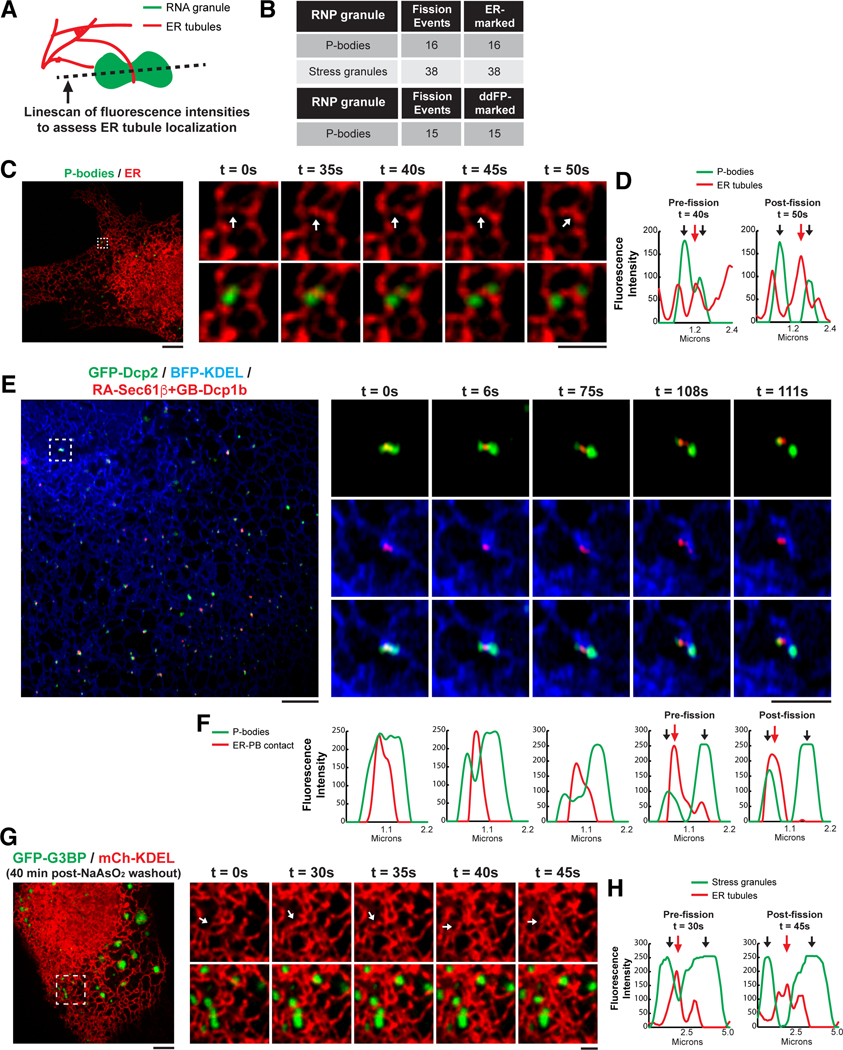
ER tubules localize to the sites of PB and stress granule fission. (**A**) Cartoon depicting line scan positioned perpendicular to RNP granule fission sites to assess ER tubule localization pre- and postfission. (**B**) Catalog of ER localization to PB and stress granule fission events as well as ddFP resolving of ER contact at PB fission events. (**C**) Representative merged images of the ER (red) labeled with mCh-KDEL and PBs (green) colabeled with GFP-Dcp1b (shown), BFP-Dcp1a, and JF646-SNAP-Dcp2 in U-2 OS cells. Insets are time-lapse images of the ER alone (top) and ER-PBs merged (bottom). Arrows highlight ER tubules positioned at PB fission sites (Movie 4). (**D**) Line scan analyses of fluorescence intensities of PBs (green) and the ER (red) pre- and postfission. Red arrows highlight ER tubules localized to the site of PB fission. (**E**) Representative images of U-2 OS cells expressing general PB (GFP-Dcp2) and ER markers (BFP-KDEL) together with the ddFP pair from Fig. 2 to resolve ER-PB contact (RA-Sec61β and GB-Dcp1b). Insets are time-lapse merged images of PBs (green) and ER-PB contact (red) (top); the ER (blue) and ER contact (red) (middle); and the ER (blue), PBs (green), and ER-PBs (red) (bottom) (Movie 5). (**F**) Line scan analyses of fluorescence intensities of PBs (green) and ER-PB contact (red) for each time point. Red arrows highlight ER tubules localized to the site of PB fission. (**G**) Representative merged images of the ER (red) labeled with mCh-KDEL and stress granules (green) labeled with GFP-G3BP in U-2 OS cells treated with 0.5 mM NaAsO_2_ for 60 min followed by 40 min of washout with 200 nM ISRIB. Arrows highlight ER tubules positioned at stress granule fission sites (Movie 6). (**H**) Line scan analyses of fluorescence intensities of stress granules (green) and the ER (red) pre- and postfission. Red arrows highlight ER tubules localized to the site of stress granule fission. In (C), (E), and (G), scale bars are 5 and 2 μm in full cell and inset images, respectively.

The fission and fusion of RNP granules, such as PBs and stress granules, have been observed in live cells (29, 39, 40). It is assumed that these events occur spontaneously given the liquid-like nature of biological condensates (16, 31). However, two observations suggest that RNP granule fission might be a regulated process within cells. First, the observation of PB fission is rare (29), which suggests that it is not energetically favorable for fission to occur spontaneously within cells. Second, the fission rate of stress granules increases dramatically during the disassembly process, which is engaged upon stress removal and restoration of translation initiation (40). Because ER contacts mediate the fission of mitochondria and early and late endosomes (13, 14), we asked whether these unconventional ER contact sites with RNP granules might also define the position of their fission. We investigated the spatiotemporal relationship between the ER and PB fission by capturing movies of U-2 OS cells expressing an ER marker (mCh-KDEL) together with PB-localized components of the mRNA decapping complex [BFP-Dcp1a, GFP-Dcp1b, and Janelia Fluor (JF)–646-SNAP-Dcp2] (Fig. 6, C and D; Movie 4; and fig. S4, A and B). To assess ER tubule localization, we measured fluorescence intensities across a line drawn along the axis of the PB perpendicular to the fission site (Fig. 6A).

We noticed that ER tubules crossed over the “constricted” neck leading up to PB fission events 100% of the time (Fig. 6, B to D, and fig. S4, A and B). To further dissect the spatiotemporal relationship between ER contact and PB fission, we captured time-lapse super-resolution movies of cells expressing the ddFP ER-PB contact system (from Fig. 2). Notably, ddFP-resolved ER contact was observed at 100% of PB fission events (Fig. 6B). Moreover, we observed fission events that displayed ER contact before the formation of a constricted neck, suggesting that the ER might contribute to constriction initiation during the fission process (Fig. 6, E and F; Movie 5; and fig. S5, C and D). This demonstrates a strict correlation between ER tubule–PB interaction at fission events analogous to the percentage of mitochondrial (88%) and endosomal fission (97%) events associated with ER tubules (13, 14).

We also asked whether ER tubules define the position of stress granule fission. Stress granules are not constitutive structures in cells but form within minutes upon exposure to stressors that inhibit translation initiation, such as NaAsO_2_. Stress granules are membraneless organelles that traffic, at least in part, by hitchhiking on endosomes or lysosomes (41) and disassemble through fission reactions after the removal of stress and upon reactivation of translation (39, 40). These dynamic properties make stress granules an ideal system for studying membraneless organelle fission and the role of the ER in this process. Thus, we visualized the spatiotemporal relationship between ER tubules and stress granule disassembly during NaAsO_2_ washout. Cells expressing ER (mCh-KDEL) and stress granule (GFP-G3BP) markers were treated with 0.5 mM NaAsO_2_ for 60 min followed by NaAsO_2_ washout for 40 min. Live imaging began at the 40-min washout time point in the presence of 200 nM integrated stress-response inhibitor (ISRIB). ISRIB expedites the stress granule disassembly process by reinitiating mRNA translation by circumventing eIF2α-phosphorylation–induced translation inhibition (38). Time-lapse movies of stress granule disassembly were captured during ISRIB treatment, and line scans were used to assess ER tubule localization during stress granule fission (Fig. 6, G and H, and fig. S5, E and F). Similar to PB fission, ER tubules rearranged across the constriction for 100% of stress granule fission events (Fig. 6, B, G, and H; Movie 6; and fig. S5, E and F).

## Discussion

Our observations presented here suggest that contact sites with the ER are not restricted to membrane-bound organelles but also occur with non–membrane-bound organelles in a functional manner. First, a large population of PBs are tethered at molecular distances to the ER in animal cells. Second, there is a relationship between PB composition and ER contact. ER contact sites with membraneless PBs share similarities in how ER MCSs interact with a membrane-bound organelle, the endosome—the percentage of endosomes tethered to the ER increases as endosomes mature. Roughly 50% of early endosomes are tethered to the ER, whereas nearly all late endosomes maintain contact with the ER (42). Thus, it is possible that the ~40 to 50% of PBs tethered to the ER represent a different PB “maturation” state from untethered PBs, such that PBs can gain contact as their composition is altered, possibly by exchanging Dcp1a and Dcp1b.

Third, our studies reveal an inverse relationship between PB biogenesis and the abundance of ER cisternae, which would be expected to have a higher ribosome density and translational capacity. In complementary experiments, the effect of ER shape on PB abundance was suppressed under conditions that inhibit translation. Given that PBs store and degrade translationally inactive mRNAs, the ability of PBs to form and disassemble in response to changes in ER translational capacity opens up the possibility that ER-PB contact sites are conduits for mRNA and/or protein exchange between the two organelles.

Lastly, our work also provides evidence that RNP granule fission can be an active process and will be mediated by ER contact sites. In particular, dynamic ER tubules defined the position of fission for two different membraneless organelles just like they do for membrane-bound organelles (13, 14). Possible machineries that could be delivered by ER tubules to drive fission of RNP granules would include protein chaperones, RNA helicases, and modification enzymes. Because it has been well documented that membraneless organelles can undergo liquid-to-solid transitions (43), it is an intriguing possibility that the ER can sense and control the physical properties of these organelles through fission. Understanding the role that the ER plays in regulating RNP granules is important because many neurodegenerative disorders are associated with age-related aggregation of RNA granule components (43, 44).

## Materials and methods

### DNA plasmids and cell lines

GFP-Dcp2, GFP-Dcp1b, and GFP-G3BP1 were a kind gift from R. Buchan (University of Arizona, Tucson, AZ). GFP-Dcp1a was generated by cloning Dcp1a from U-2 OS cDNA and inserted into XhoI/KpnI sites of the pAcGFP-C1 vector (Clontech, Mountain View, CA) and then subcloned into the BFP-C1 vector to generate BFP-Dcp1a. SNAP-Dcp2 was cloned from GFP-Dcp2 and inserted into XhoI/BamHI sites of SNAP-C1 vector. The Janelia Fluor 646 (JF-646) SNAP ligand was a kind gift from L. Lavis (Janelia Farm, Ashburn, VA). mCh-KDEL, mCh-Sec61β, Rtn4a-mCh, and BFP-KDEL were previously described (9, 13, 14). GB-NES (Addgene #61017), GA-NES (Addgene #61018), and RA-NES (Addgene #61019) vectors were a kind gift from D. Buysse and G. Odorizzi (University of Colorado, Boulder, Boulder, CO). We then generated GA-C1, GB-C1, and RA-C1 vectors by amplifying GA, GB, and RA sequences (without nuclear export sequence) from GA-NES, GB-NES, and RA-NES vectors and inserted them into the NheI/BspEI sites of the pAcGFP-C1 vector to replace the GFP-encoding sequence (Clontech, Mountain View, CA). RA-Sec61β was generated by amplifying the Sec61β-encoding sequence from GFP-Sec61β and inserting it into XhoI/KpnI sites of the RA-C1 vector. GB-Dcp1b was generated by subcloning Dcp1b from GFP-Dcp1b and inserting it into XhoI/KpnI sites of the GB-C1 vector.

The *RTN4* KO U-2 OS cell line was generated using CRISPR-Cas9 following a published protocol (45). Briefly, two guide RNAs targeting the *RTN4* gene were cloned into the lentiCRISPR v2 backbone (Addgene plasmid #52961). The two 20-nt regions that are targeted on the *RTN4* gene are CGTTCAAGTACCAGTTCGTG and GGCGCGCCCCTGATGGACTT. LentiCRISPR v2 plasmids containing two *RTN4*–targeting guide sequences (500 ng/ml each) were simultaneously transfected into U-2 OS cells using lipofectamine 3000 following the manufacturer’s protocol. Transfected cells were recovered in growth media for 24 hours and then subjected to puromycin (2 μg/ml) selection for 72 hours (with fresh puromycin every 24 hours). The surviving polycolonal population was diluted into single colonies in 96-well plates. Single clones of KO cells were verified through Western blot (Rtn4A antibody; Cell Signaling) and immunofluorescence (Rtn4A/B antibody; Santa Cruz).

### Cell culture, transfection, and drug treatments

Human osteosarcoma U-2 OS cells (ATCC-HTB 96) were tested for Mycoplasma contamination by ATCC at the time of purchase. Cells were grown in McCoy’s 5A (modified) medium supplemented with 10% fetal bovine serum (FBS) and 1% penicillin and streptomycin.

Before plating cells for imaging experiments, 35-mm glass-bottom microscope dishes (Cell Vis) were coated with 10 μg/ml of fibronectin for 5 hours at 37°C. After 5 hours, the fibronectin solution was removed, the microscope dishes were rinsed with PBS to remove excess fibronectin, and U-2 OS cells were seeded at 0.5 × 10^5^ cells/ml about 18 to 24 hours before transfection. DNA plasmid transfections were performed with 2.5 μl of lipofectamine 3000 (Invitrogen) per 1 ml of OPTI-MEM media (Invitrogen) for ~5 hours followed by a wash and replenishment with full media. Cells were imaged 18 to 24 hours after transfection in prewarmed 37°C Fluorobrite DMEM (Invitrogen) supplemented with 10% FBS and Glutamax (Invitrogen).

For all experiments, the following amounts of DNA were transfected per milliliter: 150 ng mCh-KDEL; 200 ng BFP-KDEL; 200 ng GFP-Dcp2; 200 ng SNAP-Dcp2; 200 ng GFP-Dcp1a; 200 ng BFP-Dcp1a; 200 ng GFP-Dcp1b; 200 ng GB-Dcp1b; 400 ng RA-Sec61β; 150 ng GFP-G3BP; 500 ng mCh-Sec61β; and 500 ng Rtn4a-mCh.

In ER-PB tracking during oxidative stress experiments, NaAsO_2_ was dissolved in dH_2_O to yield a 0.5 M stock solution just before treatment. U-2 OS cells expressing GFP-Dcp2 and mCh-KDEL were incubated in imaging media with 0.5 mM NaAsO_2_ for 50 min at 37°C. Cells expressing both markers were located and 2-min time-lapse movies with frames were captured every 5 s.

In stress granule disassembly experiments, GFP-G3BP and mCh-KDEL were incubated with 0.5 mM NaAsO_2_ for 1 hour. Cells were then washed and replenished with 37°C imaging media and imaged 40 min after washout with 200 nM ISRIB added into the imaging media. Two-min time-lapse movies with frames captured every 5 s permitted the capture of stress granule fission during the disassembly process.

In mRNA translation inhibition and ER stress experiments, wild-type and *RTN4* KO cells were treated with 0.5 mM NaAsO_2_ for 1 hour (oxidative stress), 200 μM puromycin for 15 min, or 1 μg/ml tunicamycin for 1 and 6 hours (ER stress) then fixed with 37°C fixative (4% paraformaldehyde, 4% sucrose in PBS) for 10 min. Cells were then permeabilized and immunolabeled with 1:200 Dcp1b monoclonal rabbit (Cell Signaling Tech) and 1:200 G3BP mouse monoclonal (Abcam) antibodies to simultaneously image PBs and stress granules, or 1:200 Edc3 mouse monoclonal (Santa Cruz) and 1:200 Calreticulin polyclonal rabbit (Abcam) antibodies to simultaneously image PBs and the ER.

### Microscopy

Imaging was performed with an inverted fluorescence microscope (TE2000-U; Nikon) equipped with an electron-multiplying charge-coupled device camera (Cascade II; Photometrics) and a Yokogawa spinning disc confocal system (CSU-Xm2; Nikon). Live-cell imaging was performed at 37°C. Images were captured with a 100× NA 1.4 oil objective and acquired using the open source microscopy software Micro-Manager. Live-cell super-resolution capture of ddFP-marked ER contact during PB fission was acquired with the Zeiss LSM 880 equipped with Airyscan detectors and 63×/1.4-NA plan Apochromat oil objective using Zeiss ZEN software.

### Immunofluorescence and analyses of PB number and ER-PB colocalization

U-2 OS cells were seeded at 0.8 × 10^5^ cells/ml on fibronection-coated coverslips and fixed, 30 hours after plating, with 37°C fixative solution (4% paraformaldehyde, 4% sucrose in PBS) for 10 min. Fixed cells were washed with PBS and permeabilized with 0.1% Triton-X100 followed by blocking with 5% normal donkey serum in PBS. Labeling of PBs and ER was achieved by incubating cells overnight at 4°C with 1:200 Edc3 mouse monoclonal (Santa Cruz) and 1:200 Calreticulin polyclonal rabbit (Abcam) antibodies in blocking serum. Cells were then washed with PBS and fluorescently labeled with donkey–anti-mouse 488 and donkey–anti-rabbit 594 secondary antibodies (Invitrogen). Cells were then washed and the nuclei labeled with Hoescht. Coverslips were mounted on microscope slides using Prolong glass resin and imaged the following day.

Using a 100× objective, Z-stack images of cells from each condition were captured within the same day under identical conditions with respect to laser intensities and exposures. Critically, the standardization of sample preparation and image capture allowed for the standardization of quantification. PB counting was accomplished by first defining the diffuse PB marker signal for each experiment. The first cell captured in the wild-type untreated condition was opened and a ROI was drawn within the cytosol, which excludes bright PB fluorescent foci, and the maximum fluorescence intensity was identified using the measure function in ImageJ. This number was then incorporated into a macros script that subtracts this fluorescence intensity from cells across all conditions followed by Yenautomated thresholding and particle analysis using the “analyze particles” plug-in in ImageJ.

The level of colocalization between PBs and ER tubules was accomplished by selecting ROIs that contained at least one PB and resolvable ER tubules. ROIs were necessary because the ER network is too dense to resolve in regions within the cell, such as the microtubule organizing center. The ROIs were cropped such that the PB was offset from the center to allow for comparison of actual images to rotated images. Segmentation of PBs was accomplished by Otsu thresholding. Segmentation of the ER was accomplished by manual thresholding owing to the broad range of ER labeling intensities throughout the cell and between ROIs. Colocalization between PBs and ER tubules was determined by calculating the Mander’s coefficient of the percentage of PBs overlapping with ER tubules (M1_PB_). To determine whether this overlap was due to chance, the ER tubule ROI was rotated 90° clockwise and the Mander’s coefficient of the percentage of PBs overlapping with the rotated ER tubule ROI was calculated (M1_90_).

### Line scan analyses of PB and stress granule fission events

For PB fission, U-2 OS cells expressing mCh-KDEL, GFP-Dcp1b, BFP-Dcp1a, and SNAP-Dcp2 were incubated with JaneliaFluor 646 in serum-free media for 15 min to conjugate a far-red fluorophore to SNAP-Dcp2, thus permitting live imaging in four channels. Time-lapse videos were acquired over the course of 2 min, with each channel captured every 5 s. Exposure times ranged between 20 and 150 ms in each channel.

The drug treatment and imaging conditions for stress granule fission are detailed above. Upon identification of PB or stress granule fission during postimaging analysis, a segmented line was drawn perpendicular to the PB or stress granule fission site through the length of the PB or stress granule. The fluorescence intensities of ER and PB or stress granule channels were measured along the length of the line for each time point and plotted. ER-marked fission events were identified by acute decreases in PB or stress granule marker fluorescence that coincided with mCh-KDEL (ER) fluorescence peaks.

## Supplementary Material

Supplemental Material **Movie 1. Live-cell tracking of the ER and PBs.** Time-lapse movie corresponding to Fig. 1C showing the ER (red) labeled with mCh-KDEL and PBs (green) labeled with GFP-Dcp2. Frames were captured every 5 s over the course of 2 min.
**Movie 2. Resolving ER-PB contact in live cells.** Time-lapse movie corresponding to Fig. 2D, inset 1, showing PBs (cyan) labeled with GFP-Dcp2, ER-PB contact (yellow) labeled with dimerization of RA-Sec61β and GB-Dcp1b, and the ER (red) labeled with BFP-KDEL. Frames were captured every 5 s over the course of 2 min.
**Movie 3. Resolving ER-PB contact in live cells.** Time-lapse movie corresponding to Fig. 2D, inset 2, showing PBs (cyan) labeled with GFP-Dcp2, ER-PB contact (yellow) labeled with dimerization of RA-Sec61β and GB-Dcp1b, and the ER (red) labeled with BFP-KDEL. Frames were captured every 5 s over the course of 2 min.
**Movie 4. ER tubules mark the site of PB fission.** Time-lapse movie corresponding to Fig. 6C showing the ER (red) labeled with mCh-KDEL and PBs simultaneously labeled with JF646-SNAP-Dcp2 (magenta), BFP-Dcp1a (blue), and GFP-Dcp1b (green). There are 5 s between each frame.
**Movie 5. ddFP system resolves the timing of ER contact during PB fission.** Time-lapse movie corresponding to Fig. 6E showing the ER (blue) labeled with BFP-KDEL, PBs (green) labeled with GFP-Dcp2, and ER contact (red) detected by RA-GB dimerization linked to RA-Sec61β and GB-Dcp1b. Images were captured every 3 s using the Zeiss LSM880 confocal laser scanning microscope with Airyscan detectors.
**Movie 6.ER tubules mark the site of stress granule fission.** Time-lapse movie corresponding to Fig. 6E showing the ER (red) labeled with mCh-KDEL and stress granules (green) labeled with GFP-G3BP. There are 5 s between each frame.

## References

[1] Staehelin LA, The plant ER: A dynamic organelle composed of a large number of discrete functional domains. Plant J. 11, 1151–1165 (1997). doi: 10.1046/j.1365-313X.1997.11061151.x; 9225461

[2] Wu H, Carvalho P, Voeltz GK, Here, there, and everywhere: The importance of ER membrane contact sites. Science 361, eaan5835 (2018). doi: 10.1126/science.aan5835; PMC6568312 30072511

[3] Vance JE, Phospholipid synthesis in a membrane fraction associated with mitochondria. J. Biol. Chem 265, 7248–7256 (1990). 2332429

[4] Rizzuto R , Close contacts with the endoplasmic reticulum as determinants of mitochondrial Ca2+ responses. Science 280, 1763–1766 (1998). doi: 10.1126/science.280.5370.1763; 9624056

[5] Kornmann B , An ER-mitochondria tethering complex revealed by a synthetic biology screen. Science 325, 477–481 (2009). doi: 10.1126/science.1175088; 19556461 PMC2933203

[6] Mesmin B , A four-step cycle driven by PI(4)P hydrolysis directs sterol/PI(4)P exchange by the ER-Golgi tether OSBP. Cell 155, 830–843 (2013). doi: 10.1016/j.cell.2013.09.056; 24209621

[7] Hua R , VAPs and ACBD5 tether peroxisomes to the ER for peroxisome maintenance and lipid homeostasis. J. Cell Biol 216, 367–377 (2017). doi: 10.1083/jcb.201608128; 28108526 PMC5294787

[8] Rocha N , Cholesterol sensor ORP1L contacts the ER protein VAP to control Rab7-RILP-p150Glued and late endosome positioning. J. Cell Biol 185, 1209–1225 (2009). doi: 10.1083/jcb.200811005; 19564404 PMC2712958

[9] Friedman JR, Dibenedetto JR, West M, Rowland AA, Voeltz GK, Endoplasmic reticulum-endosome contact increases as endosomes traffic and mature. Mol. Biol. Cell 24, 1030–1040 (2013). doi: 10.1091/mbc.e12-10-0733; 23389631 PMC3608491

[10] Zajac AL, Goldman YE, Holzbaur ELF, Ostap EM, Local cytoskeletal and organelle interactions impact molecular- motor- driven early endosomal trafficking. Curr. Biol 23, 1173–1180 (2013). doi: 10.1016/j.cub.2013.05.015; 23770188 PMC3738301

[11] Raiborg C , Repeated ER-endosome contacts promote endosome translocation and neurite outgrowth. Nature 520, 234–238 (2015). doi: 10.1038/nature14359; 25855459

[12] Knoblach B , An ER-peroxisome tether exerts peroxisome population control in yeast. EMBO J. 32, 2439–2453 (2013). doi: 10.1038/emboj.2013.170; 23900285 PMC3770948

[13] Friedman JR , ER tubules mark sites of mitochondrial division. Science 334, 358–362 (2011). doi: 10.1126/science.1207385; 21885730 PMC3366560

[14] Rowland AA, Chitwood PJ, Phillips MJ, Voeltz GK, ER contact sites define the position and timing of endosome fission. Cell 159, 1027–1041 (2014). doi: 10.1016/j.cell.2014.10.023; 25416943 PMC4634643

[15] Banani SF , Compositional control of phase-separated cellular bodies. Cell 166, 651–663 (2016). doi: 10.1016/j.cell.2016.06.010; 27374333 PMC4967043

[16] Boeynaems S , Protein phase separation: A new phase in cell biology. Trends Cell Biol. 28, 420–435 (2018). doi: 10.1016/j.tcb.2018.02.004; 29602697 PMC6034118

[17] Decker CJ, Parker R, P-bodies and stress granules: Possible roles in the control of translation and mRNA degradation. Cold Spring Harb. Perspect. Biol 4, a012286 (2012). doi: 10.1101/cshperspect.a012286; 22763747 PMC3428773

[18] Coller J, Parker R, General translational repression by activators of mRNA decapping. Cell 122, 875–886 (2005). doi: 10.1016/j.cell.2005.07.012; 16179257 PMC1853273

[19] Kilchert C, Weidner J, Prescianotto-Baschong C, Spang A, Defects in the secretory pathway and high Ca2+ induce multiple P-bodies. Mol. Biol. Cell 21, 2624–2638 (2010). doi: 10.1091/mbc.e10-02-0099; 20519435 PMC2912349

[20] Hubstenberger A , P-body purification reveals the condensation of repressed mRNA regulons. Mol. Cell 68, 144–157.e5 (2017). doi: 10.1016/j.molcel.2017.09.003; 28965817

[21] Wang C , Context-dependent deposition and regulation of mRNAs in P-bodies. eLife 7, e29815 (2018). doi: 10.7554/eLife.41300 29297464 PMC5752201

[22] Kedersha N , Dynamic shuttling of TIA-1 accompanies the recruitment of mRNA to mammalian stress granules. J. Cell Biol 151, 1257–1268 (2000). doi: 10.1083/jcb.151.6.1257; 11121440 PMC2190599

[23] Khong A , The stress granule transcriptome reveals principles of mRNA accumulation in stress granules. Mol. Cell 68, 808–820.e5 (2017). doi: 10.1016/j.molcel.2017.10.015; 29129640 PMC5728175

[24] Markmiller S , Context-dependent and disease-specific diversity in protein interactions within stress granules. Cell 172, 590–604.e13 (2018). doi: 10.1016/j.cell.2017.12.032; 29373831 PMC5969999

[25] Palade GE, A small particulate component of the cytoplasm. J. Biophys. Biochem. Cytol 1, 59–68 (1955). doi: 10.1083/jcb.1.1.59; 14381428 PMC2223592

[26] Palade G, Intracellular aspects of the process of protein synthesis. Science 189, 347–358 (1975). doi: 10.1126/science.1096303; 1096303

[27] Jan CH, Williams CC, Weissman JS, Principles of ER cotranslational translocation revealed by proximity-specific ribosome profiling. Science 346, 1257521 (2014). doi: 10.1126/science.1257521; 25378630 PMC4285348

[28] Reid DW, Chen Q, Tay AS-L, Shenolikar S, Nicchitta CV, The unfolded protein response triggers selective mRNA release from the endoplasmic reticulum. Cell 158, 1362–1374 (2014). doi: 10.1016/j.cell.2014.08.012; 25215492 PMC4163055

[29] Kedersha N , Stress granules and processing bodies are dynamically linked sites of mRNP remodeling. J. Cell Biol 169, 871–884 (2005). doi: 10.1083/jcb.200502088; 15967811 PMC2171635

[30] Brangwynne CP , Germline P granules are liquid droplets that localize by controlled dissolution/condensation. Science 324, 1729–1732 (2009). doi: 10.1126/science.1172046; 19460965

[31] Alberti S, Gladfelter A, Mittag T, Considerations and challenges in studying liquid-liquid phase separation and biomolecular condensates. Cell 176, 419–434 (2019). doi: 10.1016/j.cell.2018.12.035; 30682370 PMC6445271

[32] Ding Y , Ratiometric biosensors based on dimerization-dependent fluorescent protein exchange. Nat. Methods 12, 195–198 (2015). doi: 10.1038/nmeth.3261; 25622108 PMC4344385

[33] West M, Zurek N, Hoenger A, Voeltz GK, A 3D analysis of yeast ER structure reveals how ER domains are organized by membrane curvature. J. Cell Biol 193, 333–346 (2011). doi: 10.1083/jcb.201011039; 21502358 PMC3080256

[34] Puhka M, Joensuu M, Vihinen H, Belevich I, Jokitalo E, Progressive sheet-to-tubule transformation is a general mechanism for endoplasmic reticulum partitioning in dividing mammalian cells. Mol. Biol. Cell 23, 2424–2432 (2012). doi: 10.1091/mbc.e10-12-0950; 22573885 PMC3386207

[35] Voeltz GK, Prinz WA, Shibata Y, Rist JM, Rapoport TA, A class of membrane proteins shaping the tubular endoplasmic reticulum. Cell 124, 573–586 (2006). doi: 10.1016/j.cell.2005.11.047; 16469703

[36] Shibata Y , Mechanisms determining the morphology of the peripheral ER. Cell 143, 774–788 (2010). doi: 10.1016/j.cell.2010.11.007; 21111237 PMC3008339

[37] Walter P, Ron D, The unfolded protein response: From stress pathway to homeostatic regulation. Science 334, 1081–1086 (2011). doi: 10.1126/science.1209038; 22116877

[38] Sidrauski C, McGeachy AM, Ingolia NT, Walter P, The small molecule ISRIB reverses the effects of eIF2α phosphorylation on translation and stress granule assembly. eLife 4, e05033 (2015). doi: 10.7554/eLife.05033 25719440 PMC4341466

[39] Jain S , ATPase-modulated stress granules contain a diverse proteome and substructure. Cell 164, 487–498 (2016). doi: 10.1016/j.cell.2015.12.038; 26777405 PMC4733397

[40] Wheeler JR, Matheny T, Jain S, Abrisch R, Parker R, Distinct stages in stress granule assembly and disassembly. eLife 5, e18413 (2016). doi: 10.7554/eLife.18413; 27602576 PMC5014549

[41] Liao YC , RNA granules hitchhike on lysosomes for long-distance transport, using annexin A11 as a molecular tether. Cell 179, 147–164.e20 (2019). doi: 10.1016/j.cell.2019.08.050; 31539493 PMC6890474

[42] Friedman JR, Dibenedetto JR, West M, Rowland AA, Voeltz GK, Endoplasmic reticulum-endosome contact increases as endosomes traffic and mature. Mol. Biol. Cell 24, 1030–1040 (2013). doi: 10.1091/mbc.e12-10-0733; 23389631 PMC3608491

[43] Patel A , A liquid-to-solid phase transition of the ALS protein FUS accelerated by disease mutation. Cell 162, 1066–1077 (2015). doi: 10.1038/s41586-018-0665-2; 26317470

[44] Vogler TO , TDP-43 and RNA form amyloid-like myo-granules in regenerating muscle. Nature 563, 508–513 (2018). doi: 10.1038/s41586-018-0665-2; 30464263 PMC6324568

[45] Ran FA , Genome engineering using the CRISPR-Cas9 system. Nat. Protoc 8, 2281–2308 (2013). doi: 10.1038/nprot.2013.143; 24157548 PMC3969860

