# Supplementary material for: Endoplasmic reticulum contact sites regulate the dynamics of membraneless organelles
Source: Science. Author manuscript; Available in PMC 2023 Apr 11. (PMC10088059; doi:10.1126/science.aay7108)
Supplement: Supplemental Material — Movie 1. Live-cell tracking of the ER and PBs. Time-lapse movie corresponding to Fig. 1C showing the ER (red) labeled with mCh-KDEL and PBs (green) labeled with GFP-Dcp2. Frames were captured every 5 s over the course of 2 min. Movie 2. Resolving ER-PB contact in live cells. Time-lapse movie corresponding to Fig. 2D, inset 1, showing PBs (cyan) labeled with GFP-Dcp2, ER-PB contact (yellow) labeled with dimerization of RA-Sec61β and GB-Dcp1b, and the ER (red) labeled with BFP-KDEL. Frames were captured every 5 s over the course of 2 min. Movie 3. Resolving ER-PB contact in live cells. Time-lapse movie corresponding to Fig. 2D, inset 2, showing PBs (cyan) labeled with GFP-Dcp2, ER-PB contact (yellow) labeled with dimerization of RA-Sec61β and GB-Dcp1b, and the ER (red) labeled with BFP-KDEL. Frames were captured every 5 s over the course of 2 min. Movie 4. ER tubules mark the site of PB fission. Time-lapse movie corresponding to Fig. 6C showing the ER (red) labeled with mCh-KDEL and PBs simultaneously labeled with JF646-SNAP-Dcp2 (magenta), BFP-Dcp1a (blue), and GFP-Dcp1b (green). There are 5 s between each frame. Movie 5. ddFP system resolves the timing of ER contact during PB fission. Time-lapse movie corresponding to Fig. 6E showing the ER (blue) labeled with BFP-KDEL, PBs (green) labeled with GFP-Dcp2, and ER contact (red) detected by RA-GB dimerization linked to RA-Sec61β and GB-Dcp1b. Images were captured every 3 s using the Zeiss LSM880 confocal laser scanning microscope with Airyscan detectors. Movie 6.ER tubules mark the site of stress granule fission. Time-lapse movie corresponding to Fig. 6E showing the ER (red) labeled with mCh-KDEL and stress granules (green) labeled with GFP-G3BP. There are 5 s between each frame. [file NIHMS1874219-supplement-Supplemental_Material.pdf]

Science
AAAS

Supplementary Materials for

ER contact sites regulate the dynamics of membrane-less organelles

Authors: Jason E. Lee[1], Peter I. Cathey[1,3], Haoxi Wu[1], Roy Parker[2,3], Gia K. Voeltz*[1,3].

Correspondence to: Gia.Voeltz@Colorado.edu

This PDF file includes:

Materials and Methods
Figs. S1 to S5

Materials and Methods

DNA plasmids and cell lines: GFP-Dcp2, GFP-Dcp1b and GFP-G3BP1 were gifts from Ross Buchan. GFP-Dcp1a was generated by cloning Dcp1a from U-2 OS cDNA and inserted into XhoI/KpnI sites of the pAcGFP-C1 (Clontech, Mountain View, CA) and then subcloned into the BFP-C1 vector to generate BFP-Dcp1a. SNAP-Dcp2 was cloned from GFP-Dcp2 and inserted into XhoI/BamHI sites of SNAP-C1 vector. mCh-KDEL, mCh-Sec61β, Rtn4a-mCh, BFP-KDEL were previously described *(9, 13, 14)*. GB-NES (Addgene #61017), GA-NES (Addgene #61018), RA-NES (Addgene #61019) vectors were gifts from Dalton Buysse and Greg Odorizzi. We then generated GA-C1, GB-C1, RA-C1 vectors were generated by amplifying GA, GB, RA sequences (without Nuclear Export Sequence) from GA-NES, GB-NES, RA-NES vectors and inserted into the NheI/BspEI sites of pAcGFP-C1 vector to replace the GFP-encoding sequence (Clontech, Mountain View, CA). RA-Sec61β was generated by amplifying Sec61β-encoding sequence from GFP-Sec61β and inserting into XhoI/KpnI sites of RA-C1 vector. GB-Dcp1b was generated by subcloning Dcp1b from GFP-Dcp1b and inserting into XhoI/KpnI sites of GB-C1 vector.

RTN4 knockout U-2 OS cell line was generated using CRISPR-Cas9 following a published protocol (Ran FA *et al.* Nat Protoc. 2013 Pubmed ID: 24157548). Briefly, two guide RNAs targeting the RTN4 gene were cloned into lentiCRISPR v2 backbone (Addgene plasmid #52961). The two 20nt regions that are targeted on the RTN4 gene are CGTTCAAGTACCAGTTCGTG and GGCGCGCCCCTGATGGACTT. LentiCRISPR v2 plasmids containing two RTN4 targeting guide sequences (500 ng/mL each) are simultaneously transfected into U-2 OS cells using Lipofectamine 3000 following manufacturer's protocol. Transfected cells are recovered in growth media for 24 hours and then subject to puromycin (2 μg/mL) selection for 72 hours (with fresh puromycin every 24 hours). Surviving polycolonal population is diluted into single colony in 96 well plates. Single clones of knockout cells are verified through western blot (Rtn4A antibody; Cell Signaling) and immunofluorescence (Rtn4A/B antibody; Santa Cruz).

Cell culture, transfection, and drug treatments: Human osteosarcoma U-2 OS cells (ATCC-HTB 96) were tested for Mycoplasma contamination by ATCC at the time of purchase. Cells were grown in McCoy's 5A (Modified) medium supplemented with 10% fetal bovine serum (FBS) and 1% penicillin/streptomycin.

Prior to plating cells for imaging experiments, 35 mm glass-bottom microscope dishes (Cell Vis) were coated with 10 μg/mL of fibronectin for 5 hours at 37°C. After 5 hours, the fibronectin solution was removed, the microscope dishes were rinsed with PBS to remove excess fibronectin, and U2OS cells were seeded at 0.5×10^5 cells/mL about 18-24 hours prior to transfection. DNA plasmid transfections were performed with 2.5 μL of Lipofectamine 3000 (Invitrogen) per 1 mL of in OPTI-MEM media (Invitrogen) for ~5 hours followed by a wash and replenishment with full media. Cells were imaged 18-24 after transfection in pre-warmed 37°C Fluorobrite DMEM (Invitrogen) supplemented with 10% FBS and Glutamax (Invitrogen).

For all experiments, the following amounts of DNA were transfected per mL: 150 ng mCh-KDEL; 200 ng BFP-KDEL; 200 ng GFP-Dcp2; 200 ng SNAP-Dcp2; 200 ng GFP-Dcp1a; 200 ng BFP-Dcp1a; 200 ng GFP-Dcp1b; 200 ng GB-Dcp1b; 400 ng RA-Sec61β; 150 ng GFP-G3BP; 500 ng mCh-Sec61β; 500 ng Rtn4a-mCh.

In ER-PB tracking during oxidative stress experiments, sodium arsenite ($NaAsO_2$) was dissolved in dH_2O to yield a 0.5 M stock solution just prior to treatment. U-2 OS cells expressing GFP-Dcp2

and mCh-KDEL were incubated in imaging media with 0.5 mM $NaAsO_2$ for 50 minutes at 37°C. Cells expressing both markers were located and 2-minute time-lapse movies with frames captured every 5 seconds.

In stress granule disassembly experiments, GFP-G3BP and mCh-KDEL were incubated with 0.5 mM $NaAsO_2$ for 1 hour. Cells were then washed and replenished with 37°C imaging media and imaged 40 minutes after washout with 200 nM integrated stress response inhibitor (ISRIB) added into the imaging media. 2-minute time-lapse movies with frames captured every 5 seconds permitted the capture of stress granule fission during the disassembly process.

In mRNA translation inhibition and ER stress experiments, *wild-type* and *RTN4* KO cells were treated with 0.5 mM $NaAsO_2$ for 1 hour (oxidative stress), 200 μM puromycin for 15 minutes, or 1 μg/mL tunicamycin for 1 and 6 hours (ER stress) then fixed with 37°C fixative (4% paraformaldehyde, 4% sucrose in PBS) for 10 minutes. Cells were then permeabilized immunolabeled with 1:200 Dcp1b monoclonal rabbit (Cell Signaling Tech) and 1:200 G3BP mouse monoclonal (Abcam) antibodies to simultaneously image PBs and stress granules, or 1:200 Edc3 mouse monoclonal (Santa Cruz) and 1:200 Calreticulin polyclonal rabbit (Abcam) antibodies to simultaneously image PBs and the ER.

Microscopy: Imaging was performed with an inverted fluorescence microscope (TE2000-U; Nikon) equipped with an electron-multiplying charge-coupled device camera (Cascade II; Photometrics) and a Yokogawa spinning disc confocal system (CSU-Xm2; Nikon). Live-cell imaging was performed at 37°C. Images were captured with a 100x NA 1.4 oil objective and acquired using the open source microscopy software, Micro-Manager. Live-cell super-resolution capture of ddFP-marked ER contact during PB fission were acquired the Zeiss LSM 880 equipped with Airyscan detectors and 63×/1.4-NA plan Apochromat oil objective using Zeiss ZEN software.

Immunofluorescence and analyses of P-body number and ER-P-body colocalization: U-2 OS cells were seeded at 0.8×10^5 cells/mL on fibronection-coated coverslips and fixed, 30 hours after plating, with 37°C fixative solution (4% paraformaldehyde, 4% sucrose in PBS) for 10 minutes. Fixed cells were washed with PBS and permeabilized with 0.1% Triton-X100 followed by blocking with 5% normal donkey serum in PBS. Labeling of P-bodies and ER was achieved by incubating cells overnight at 4C with 1:200 Edc3 mouse monoclonal (Santa Cruz) and 1:200 Calreticulin polyclonal rabbit (Abcam) antibodies in blocking serum. Cells were then washed with PBS and fluorescently labeled with donkey-anti-mouse 488 and donkey-anti-rabbit 594 secondary antibodies (Invitrogen). Cells were then washed and the nuclei labeled with Hoescht. Coverslips were mounted on microscope slides using Prolong glass resin and imaged the following day.

Using a 100x objective, Z-stack images of cells from each condition were captured within the same day under the identical conditions with respect to laser intensities and exposures. Critically, the standardization of sample preparation and image capture allowed for the standardization of quantification. P-body counting was accomplished by first defining the diffuse PB marker signal for each experiment. The first cell captured in *wild-type* untreated condition was opened and an ROI was drawn within the cytosol, which excludes bright PB fluorescent foci, and the maximum fluorescence intensity was identified using the measure function in ImageJ. This number was then incorporated into a macros script that subtracts this fluorescence intensity from cells across all conditions followed by Yen-automated thresholding and particle analysis using the *analyze particles* plug-in in ImageJ.

The level of colocalization between P-bodies and ER tubules was accomplished by selecting ROIs, which contained at least one P-body and resolvable ER tubules. ROIs were necessary because the ER network is too dense to resolve in regions within the cell, such as the microtubule organizing center. The ROIs were cropped such that the P-body was offset from the center to allow for comparison of actual images to rotated images. Segmentation of P-bodies was accomplished by Otsu thresholding. Segmentation of the ER was accomplished by manual thresholding due to the broad range of ER labeling intensities throughout the cell and between ROIs. Colocalization between P-bodies and ER tubules was determined by calculating the Mander's coefficient of the percentage P-bodies overlapping with ER tubules ($M1_{PB}$). To determine whether this overlap was due to chance, the ER tubule ROI was rotated 90 degrees clockwise and the Mander's coefficient of the percentage P-bodies overlapping with the rotated ER tubule ROI was calculated ($M1_{90}$).

Linescan analyses of P-body and stress granule fission events: For P-body fission, U-2 OS cells expressing mCh-KDEL, GFP-Dcp1b, BFP-Dcp1a, SNAP-Dcp2 were incubated with JaneliaFluor 646 in serum free media for 15 minutes to conjugate a far-red fluorophore to SNAP-Dcp2, thus permitting live imaging in 4-channels. Time-lapse videos were acquired over the course of 2 minutes with each channel captured every 5 seconds. Exposure times ranged between 20–150 ms in each channel.

The drug treatment and imaging conditions for stress granule fission are detailed above. Upon identification of P-body or stress granule fission during post-imaging analysis, a segmented line was drawn perpendicular to the P-body/stress granule fission site through the length of the P-body/stress granule. The fluorescence intensities of ER and P-body/stress granule channels were measured along the length of the line for each time point and plotted. ER-marked fission events were identified by acute decreases in P-body/stress granule marker fluorescence that coincided with mCh-KDEL (ER) fluorescence peaks.

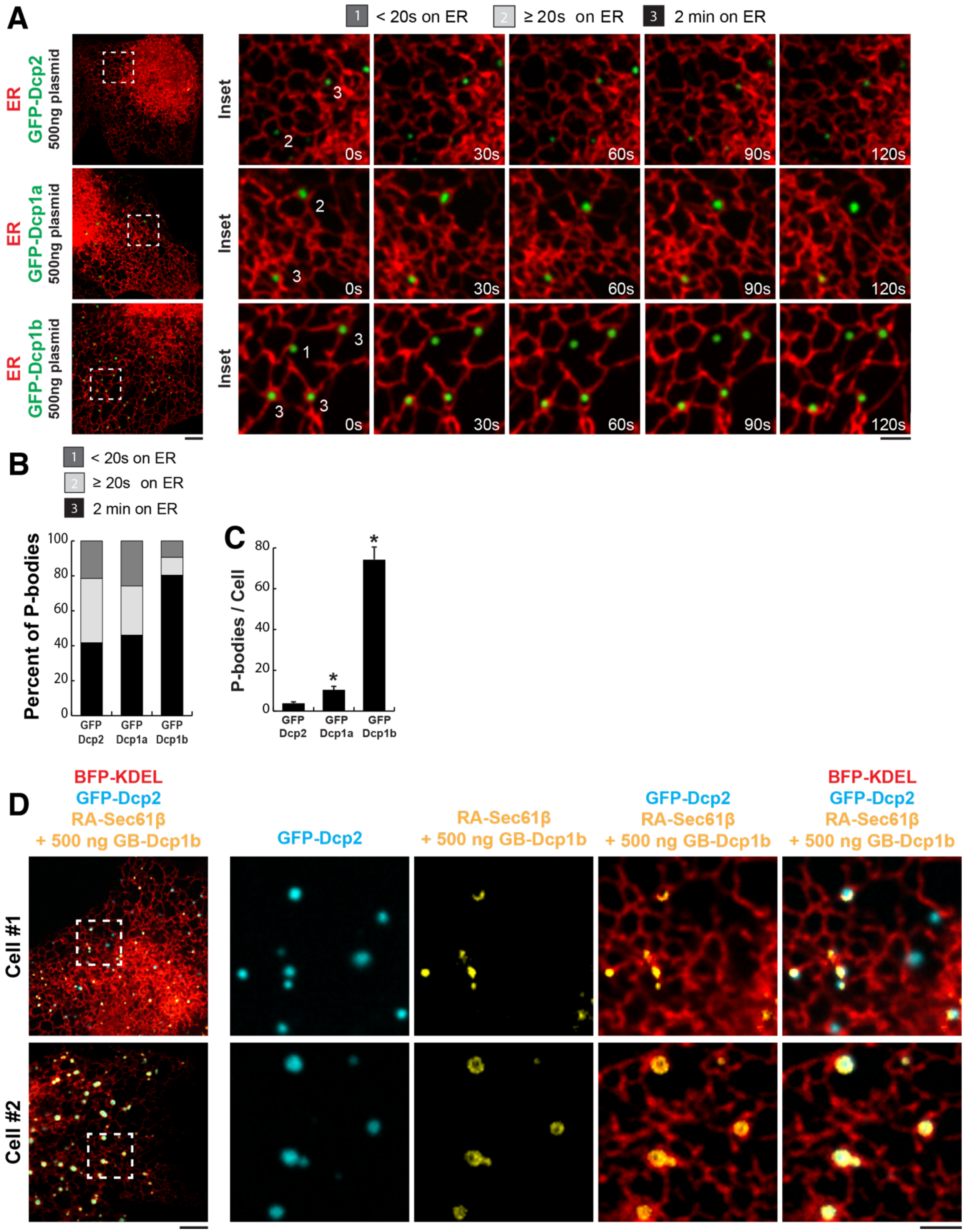

Fig. S1. Overexpression of Dcp1b results in an increase in P-body biogenesis and ER-P-body contact. (A) Representative merged images U-2 OS cells transiently transfected with 500 ng of

plasmid encoding for various components of the mRNA decapping complex (GFP-Dcp2, GFP-Dcp1a or GFP-Dcp1b in green) together with an ER marker (mCh-KDEL in red). Insets show movement of the two organelles through space and time over a two-minute time-lapse with frames captured every five seconds. P-bodies highlighted with a "1" tracked with ER tubules for less than four consecutive frames; whereas P-bodies highlighted with a "3" tracked with ER tubules for the entire time-lapse. 30 cells were imaged for each condition from three biological replicates and quantified for (B) the degree of association between the ER and P-bodies, and (C) the mean number of P-bodies per cell. (D) Representative images of two U-2 OS cells transiently transfected with 500 ng GB-Dcp1b plasmid (compared to 200 ng in Fig. 2C-E), RA-Sec61β, GFP-Dcp2 (cyan), BFP-KDEL (red). ER-P-body contact (yellow) is resolved by dimerization of RA-Sec61β and GB-Dcp1b. Insets from the two cells show examples of extensive ER-P-body contact (yellow). (A, D) Scale bars = 5 μm and 2 μm in full cell and inset images, respectively.

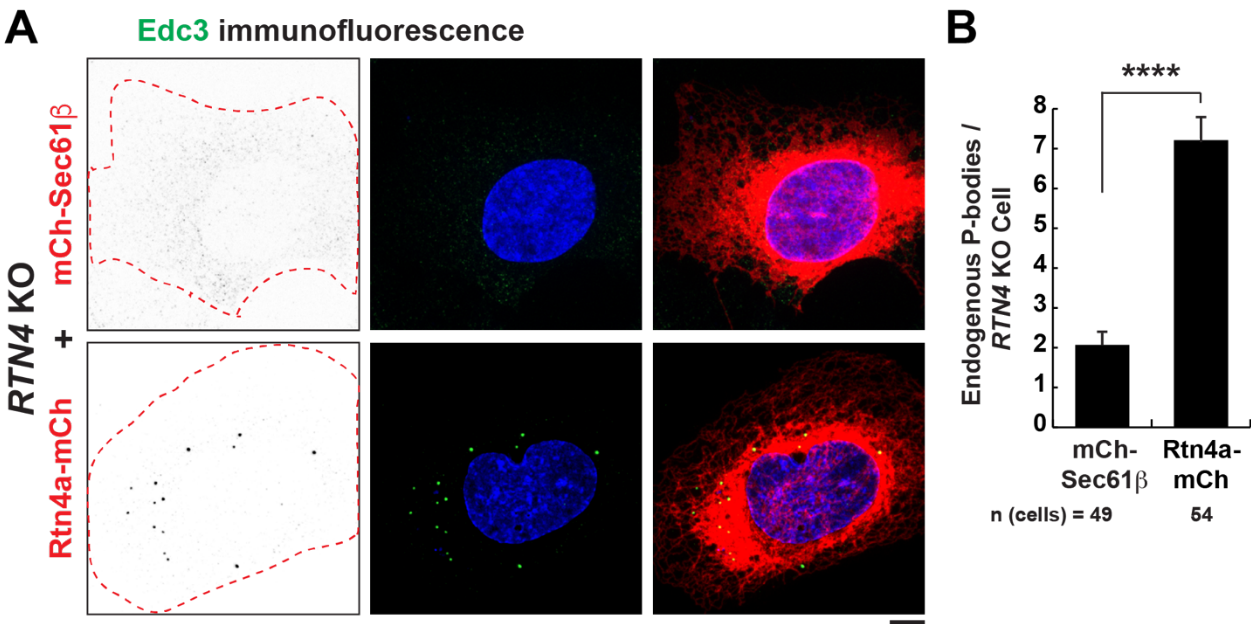

Fig. S2. The loss of PB biogenesis in *RTN4* KO cells can be rescued by exogenous expression of Rtn4a. (A) Representative images of Edc3 immunofluorescence studies performed in *RTN4* KO U-2 OS cells transiently transfected with mCh-Sec61β or Reticulon-4a (Rtn4a)-mCh. Scale bars = 5 µm. (B) The mean number of endogenous P-bodies were quantified in *RTN4* KO U-2 OS cells exogenously expressing mCh-Sec61β or Rtn4a-mCh from three biological replicates. Statistical significance was determined by Student's T-test.

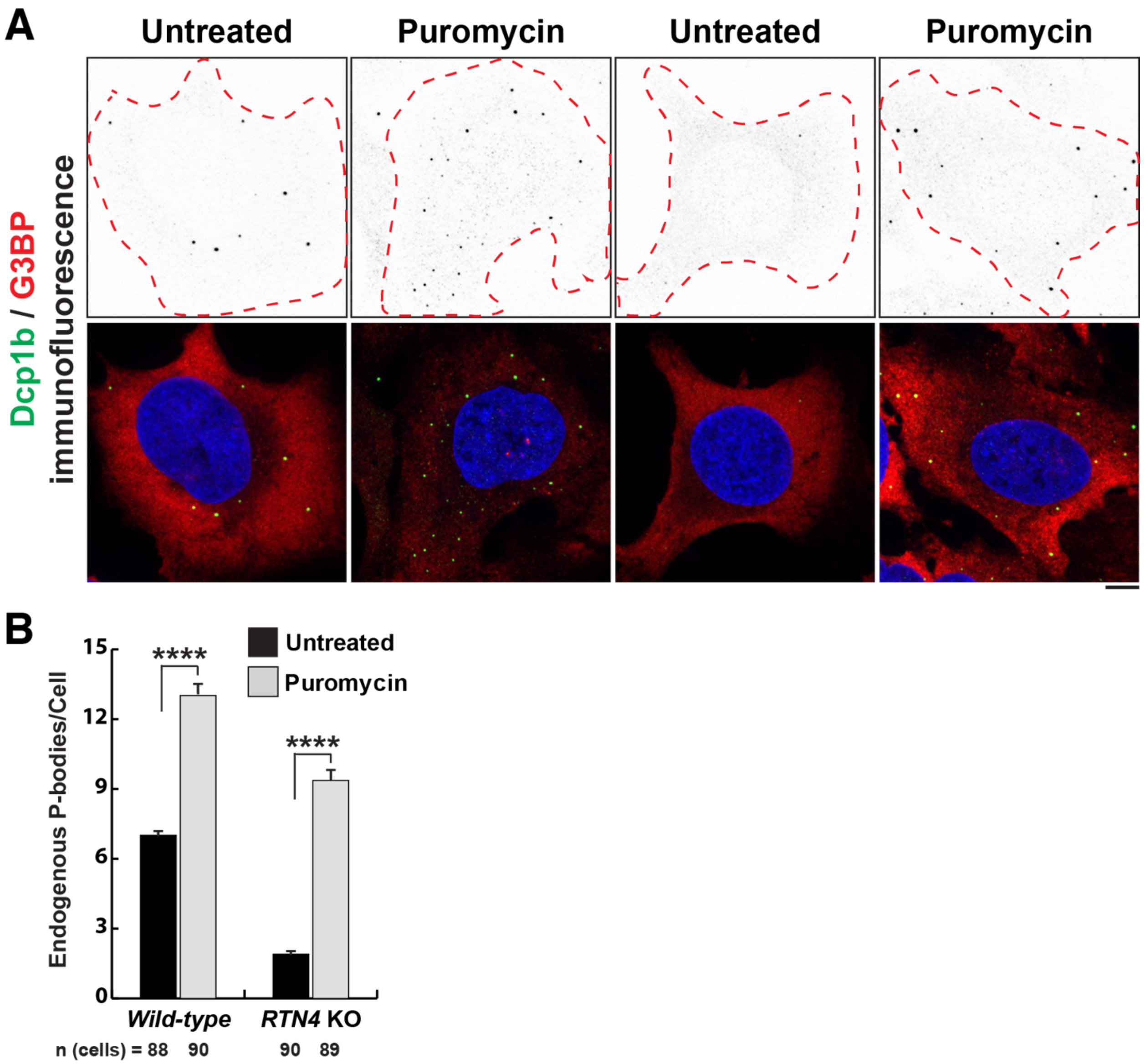

Fig. S3. The relationship between PB biogenesis, ER shape, and puromycin-induced mRNA translation inhibition. (A) Representative images of Dcp1b (P-body marker) and G3BP (stress granule marker) immunofluorescence studies were performed in *wild-type* and *RTN4* KO U-2 OS cells treated with 200 µM puromycin for 15 minutes. (B) The mean number of endogenous P-bodies were quantified from three biological replicates. Statistical significance was determined by one-way ANOVA with multiple comparisons. Scale bar = 5 µm.

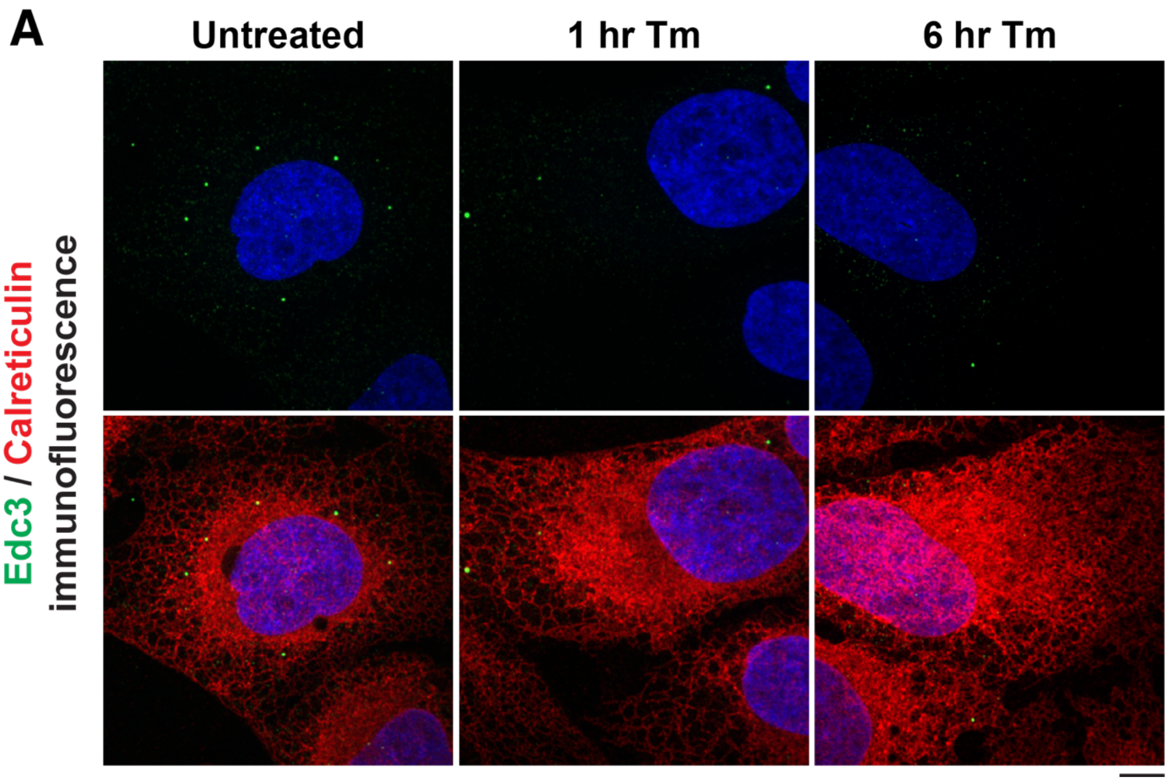

Fig. S4. Tunicamycin induced ER stress and an expansion of cisternal ER. (A) Representative images of Edc3 and calreticulin (ER marker) immunofluorescence studies were performed in U-2 OS cells that were either left untreated or treated with 1 μg/mL tunicamycin for 1 and 6 hours (hr). Scale bars = 5 μm.

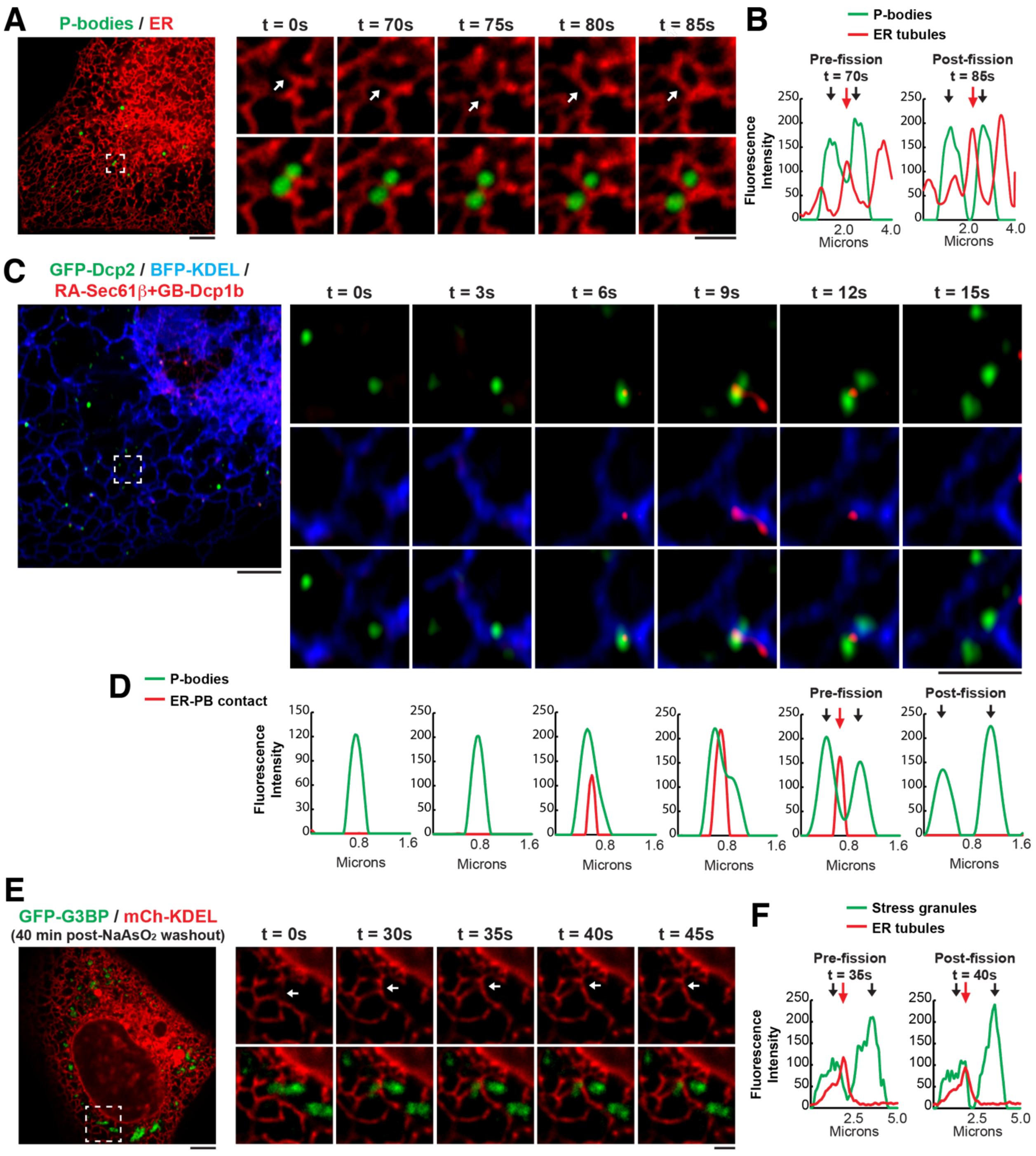

Fig. S5. ER tubules localize to the sites of P-body and stress granule fission. (A) Representative merged images of the ER (red) labeled with mCh-KDEL and P-bodies (green) co-labeled with GFP-Dcp1b (pictured), BFP-Dcp1a, JF646-SNAP-Dcp2 in U-2 OS cells. Insets are time-lapsed images of the ER alone (top) and ER-P-bodies merged (bottom). Arrows highlight ER tubules positioned at P-body fission sites. (B) Line-scan analyses of fluorescence intensities of P-bodies (green) and the ER (red) pre-fission and post-fission. Red arrows highlight ER tubules localized to the site of P-body fission. (C) Representative images of U-2 OS cells expressing general P-body (GFP-Dcp2) and ER markers (BFP-KDEL) together with the ddFP pair from Fig. 2 to resolve ER-

PB contact (RA-Sec61β and GB-Dcp1b). Insets are time-lapsed merged images of (top) P-bodies (green) and ER-PB contact (red), (middle) the ER (blue) and ER contact (red), (bottom) the ER (blue), P-bodies (green) and ER-PBs (red) together. (D) Line-scan analyses of fluorescence intensities of P-bodies (green) and ER-PB contact (red) for each time-point. Red arrows highlight ER tubules localized to the site of P-body fission. (E) Representative merged images of the ER (red) labeled with mCh-KDEL and stress granules (green) labeled with GFP-G3BP in U-2 OS cells treated with 0.5mM $NaAsO_2$ for 60 minutes followed by 40 minutes of washout with the integrated stress response inhibitor (200 nM ISRIB). Insets are time-lapsed images of the ER alone (top) and ER-stress granules merged (bottom) leading up to and following stress granule fission. Arrows highlight ER tubules positioned at stress granule fission sites. (F) Linescan analyses of fluorescence intensities of stress granules (green) and the ER (red) pre-fission and post-fission. Red arrows highlight ER tubules localized to the site of stress granule fission. (A, C, E) Scale bars = 5 μm and 2 μm in full cell and inset images, respectively.
